# Epidermal cell cultures from white and green sturgeon (*Acipenser transmontanus and medirostris*): Expression of TGM1-like transglutaminases and CYP4501A

**DOI:** 10.1371/journal.pone.0265218

**Published:** 2022-03-16

**Authors:** Noreen Karim, Lo-Wei Lin, Joel P. Van Eenennaam, Nann A. Fangue, Andrea D. Schreier, Marjorie A. Phillips, Robert H. Rice

**Affiliations:** 1 Department of Environmental Toxicology, University of California, Davis, California, United States of America; 2 Department of Animal Science, University of California, Davis, California, United States of America; 3 Department of Wildlife, Fish and Conservation Biology, University of California, Davis, California, United States of America; UNITED STATES

## Abstract

Using a system optimized for propagating human keratinocytes, culture of skin samples from white and green sturgeons generated epithelial cells capable of making cross-linked protein envelopes. Two distinct forms of TGM1-like mRNA were molecularly cloned from the cells of white sturgeon and detected in green sturgeon cells, accounting for their cellular envelope forming ability. The protein translated from each displayed a cluster of cysteine residues resembling the membrane anchorage region expressed in epidermal cells of teleosts and tetrapods. One of the two mRNA forms (called A) was present at considerably higher levels than the other (called B) in both species. Continuous lines of white sturgeon epidermal cells were established and characterized. Size measurements indicated that a substantial fraction of the cells became enlarged, appearing similar to squames in human epidermal keratinocyte cultures. The cultures also expressed CYP1A, a cytochrome P450 enzyme inducible by activation of aryl hydrocarbon receptor 2 in fish. The cells gradually improved in growth rate over a dozen passages while retaining envelope forming ability, TGM1 expression and CYP1A inducibility. These cell lines are thus potential models for studying evolution of fish epidermis leading to terrestrial adaptation and for testing sturgeon sensitivity to environmental stresses such as pollution.

## Introduction

Aquatic organisms are in decline worldwide, and many desirable species, particularly those in estuaries impacted by human civilization, are threatened or endangered. In addition to overfishing, this phenomenon is attributable to loss of habitat and its degradation by pollution. To permit remediating and restoring the remaining habitat, a great need exists to identify pollutants of most concern to support regulatory action. However, traditional toxic testing is slow, costly and can lead to sacrificing considerable numbers of individual animals. In contrast, cell lines provide important models for physiology, virology, biotechnology and toxicology that, once established, permit testing without further loss of live individuals. This factor is highly relevant to sturgeon species, most of which are threatened or endangered. Two species of conservation concern are the evolutionary octoploids, white sturgeon (*Acipenser transmontanus*) and green sturgeon (*Acipenser medirostris*), both of which inhabit coastal regions and large river systems along the West Coast of North America [1, 2]. The green sturgeon Southern Distinct Population Segment (SDPS) and Kootenai River white sturgeon population are listed under the US Endangered Species Act as threatened and endangered, respectively [3, 4]. The SDPS green sturgeon and the Sacramento-San Joaquin population of white sturgeon, listed as of “special concern” in the state of California [5], both use the San Francisco Estuary system at various points in their life cycles. The San Francisco Estuary is impacted by substantial levels of many legacy and current pollutants known to be hazardous to aquatic species (including metals, pesticides, halogenated aromatic hydrocarbons, pharmaceuticals), due to runoff from urban and agricultural sources, as well as many pollutants only recently identified [6, 7].

Among the many classes of pollutants to which fish such as sturgeon are exposed, polycyclic and halogenated aromatic hydrocarbons are widely encountered and known to be deleterious. Fish species differ by >100 fold in sensitivity to these compounds, where most damage occurs during larval development [8]. Relative species sensitivity can be estimated from responsiveness to the highly potent prototypical compound, 2,3,7,8-tetrachlorodibenzo-p-dioxin (TCDD). This pollutant induces the enzyme CYP1A that helps degrade and clear xenobiotics but that also activates some substrates to electrophiles that damage cellular macromolecules. Sturgeon have two forms of aryl hydrocarbon receptor (AHR1, AHR2), where the latter is responsible for induction of CYP1A and most of the myriad deleterious effects of TCDD and similar ligands [9, 10]. TCDD can act as an endocrine disruptor, for example, by suppressing estrogenic responsiveness (vitellogenin synthesis) in white sturgeon [11].

Although cell lines have been derived from many fish species, especially those available in aquaculture, generally they are of mesenchymal origin. However, epithelial cell lines are derivable by a method found successful with fish integument [12]. The culture system uses a feeder layer of lethally irradiated mouse 3T3 embryonic fibroblasts [13]. It specifically selected for growth of keratinocytes from a mouse teratoma in its first use [14] and has since been optimized to promote growth of human epidermal keratinocytes [15]. Fortuitously, when supplemented with the rho kinase inhibitor Y-27632 [16], this system selected for growth of tilapia lip epithelial cells that we could not propagate otherwise [17].

In addition to its potential use for ecotoxicology studies, the present cultures of an epithelial cell type from sturgeon epidermis provided an initial opportunity to characterize the cellular properties. During evolution, the emergence of aquatic species onto land involved multiple adaptations. Recent genome sequencing and transcriptional analysis has revealed traits related to terrestrial adaptation in ancient lobe-finned fish lineages, of which lungfish is the closest relative to tetrapods. These traits involve the respiratory and nervous systems and limb and cardiac development [18–20]. Some of these traits, such as a latent potential for limb development, are also found in teleosts [21]. Dramatic morphological and transcriptional changes in the skin of modern amphibious teleosts upon exposure to air could have facilitated ancient terrestrial adaptation [22]. However, the epidermis of ancient fish lineages such as sturgeon appears quite different from that of tetrapods [23], where lineages leading to *Actinopterygians* and tetrapods diverged >450 million years ago [18, 20]. Thus, characterization of the cultured epidermal cells might shed light on repurposing of features in the sturgeon lineage during evolution of tetrapod epidermis.

In addition to providing a barrier to the environment, a function of epidermis in tetrapods such as mammals is to regulate evaporative water loss [24]. To that end, keratinocyte transglutaminase (TGM1) in human epidermis plays a critical role by cross-linking a multitude of proteins to form the cross-linked envelope [25]. This characteristic feature of mature corneocytes provides a scaffold to which lipid is covalently attached, forming a barrier limiting transepidermal evaporation [26]. Although fish epidermis generally lacks a protective cornified outer layer, stratified external epithelia are observed in certain teleost tissues, such as in the lip of tilapia and, more dramatically, in breeding tubercles and contact organs of some teleosts [27]. Moreover, the skin of the Yantze sturgeon (*A*. *dabryanus*) displays keratinized spines [23] and keratinocyte-like cells, identified by numerous intermediate filaments and desmosomes, have been reported in integument of larval lake sturgeon (*A*. *fulvescens*) [28].

We pursued the hypothesis that adaptation of stratified epithelia of fish for terrestrial survival involved participation of TGM1-like protein cross-linking. Analysis of cultured epithelial cells from *O*. *mossambicus* (tilapia) revealed two related TGM1-like enzymes capable of forming envelope structures, and database searches indicate this isozyme class is common in teleosts [17]. Although other types of transglutaminase were found, TGM1 was not identified previously by database searching in more ancient aquatic species. However, a high level of isopeptide cross-linking is found in envelope structures at the periphery of cells in the horny teeth of hagfish [29], suggesting that the ancient agnathan lineage expresses a TGM1-like enzyme. Present work now identifies such an enzyme in sturgeon, a lineage that diverged from teleosts some 400 million years ago [18].

## Materials and methods

### Cell culture

Pieces of skin from the flank, ventral surface and edge of the protruding mouth of four white sturgeon were excised, placed in culture medium, transported to the laboratory at ambient temperature and used to generate explant cultures as previously described for tilapia [12]. Three samples originated from normal octoploid white sturgeon while one was taken from a spontaneous autopolyploid, an individual possessing an additional four copies of the maternal genome (dodecaploid) [30]. Cultures were maintained at room temperature (24–26°C) in a 5% CO_2_ atmosphere with a feeder layer of lethally irradiated 3T3 fibroblasts in Dulbecco-Vogt modified Eagle’s medium (high glucose) supplemented with 5% fetal bovine serum, 0.4 μg/ml hydrocortisone, 10 ng/ml epidermal growth factor, 10 μM Y27632 and 10 μM ciprofloxacin. Fibroblasts were occasionally observed, but the epithelial cells attached more tightly to the dishes and outcompeted them. The medium was changed first after 1–2 days and then at 4 day intervals. Cultures were split 1:2 with trypsin and EDTA when they reached confluence using a 3T3 feeder layer previously permitted to attach to the dishes at 37°C. In these experiments, cells from octoploid and dodecaploid white sturgeon grew equally well.

Fresh samples of ventral surface skin from one green sturgeon (octoploid), obtained as above, were minced with scissors and incubated in a plastic centrifuge tube (held horizontally) with culture grade trypsin for 45 min at room temperature with occasional swirling. After the tube was held vertically for several seconds to permit the large pieces of skin to settle, the cells released into the supernatant were drawn off, recovered by centrifugation and, after resuspension in serum containing medium, added to culture dishes with pre-attached 3T3 feeder layers. This digestion and recovery process was repeated two more times, after which the remaining tissue fragments were added to other dishes with a feeder layer.

### Cell harvesting and envelope induction

When the epithelial cell outgrowths or colonies reached diameters of ≈1 cm or became confluent, they were dissolved in Trizol and stored at -80°C for subsequent real time PCR analysis or cDNA cloning. Alternatively, such cultures were incubated for 16 hr in serum free medium containing 0.1 mg/ml ionophore X537A. Floating and attached cells were treated together with a solution 2% in sodium dodecyl sulfate, 20 mM in Tris buffer (pH 7.5), and 20 mM in dithiothreitol (DTT) for at least an hour before visualization by phase contrast microscopy [31].

### Cloning, sequencing and PCR

A partial male octoploid white sturgeon genome sequence was obtained in a pilot study (Schreier, unpublished) using the 10X Genomics Chromium platform and two lanes of Illumina HiSeq 4000 sequencing assembled by Supernova v.2.0.0 [32]. The sequence was interrogated using tblastn with the known sequences (called A and B) of the two TGM1s from sterlet sturgeon (*Acipenser ruthenus*) [33]. Matching regions of the white sturgeon genome were scanned for translated regions and compiled; the sequence obtained covered exons 3–14 for each form (S1 Table in S1 File). The remainder of the 5’-end was cloned using a TGM1-specific primer to synthesize cDNA from cultured cell RNA. The cDNA was tailed with oligo dC using terminal transferase, and PCR was conducted with oligo dG and a nested Tgm1-specific primer. Amplification of 5’ ends was performed using a 5’ RACE system for rapid amplification of cDNA ends (Thermo Fisher Scientific). PCR products (0.7–1 kb) were recovered and cloned using a TOPO TA Cloning kit for subcloning (Thermo Fisher Scientific). After confirmation by their restriction fragmentation patterns, the cloned products were submitted to the University of California Davis DNA Technologies Core for sequencing. In parallel, the transcribed white sturgeon genomic DNA sequence from exons 3–15 was submitted to Applied Biosystems (Thermo Fisher) for Custom TaqMan Gene Expression Assay synthesis. Real time PCR was conducted on a BioRad CFX96 C1000 Touch Thermal Cycler. Primer sequences for the various steps are given in S2 Table in S1 File. Ct values of the A forms were 18–22 for white sturgeon (most measurements from 2 or 3 independent cultures) and 16–20 for green sturgeon (two independent cultures).

### Ethoxyresorufin-O-deethylase (EROD) assay

Cultures grown to confluence in 12 well plates were incubated overnight with the AHR ligand TCDD with or without AHR inhibitors. The cells were then treated with 7-ethoxyresorufin (4 μM) in serum free medium. After a 2 hr incubation, the serum free medium was harvested and its content of 7-hydroxy-resorufin, the dealkylated product, was measured by fluorescence (excitation at 560 nm, emission at 600 nm) using a SpectraMax iD3 Multi-Mode microplate reader (Molecular Devices, San Jose, USA). 7-ethoxyresorufin has been a useful substrate in measuring cytochrome P450 1A activity in intact mouse epidermal cells [34] and serves an analogous function to measurement of benzo(a)pyrene metabolites in cultured human and rodent epidermal cells [35].

### Cell size distribution

Cultures were trypsinized, recovered by centrifugation, resuspended in medium and an aliquot (100 μl) diluted with 20 ml of saline solution from which 0.1 ml aliquots were analyzed electronically at low cell densities to avoid coincidence counting. The size distributions were determined using a Beckman Coulter Multisizer3 to measure cell electrical resistances, proportional to their volumes, from which the diameters are calculated assuming spherical shapes. Although the attached cells in culture resemble squames, this is a good approximation because they become rounded up as a result of trypsin treatment, analogous to human epidermal keratinocytes [36].

### Ethics statement

This study was carried out using only culled sturgeon (not subjected to experimental treatments). Until sacrifice, they were maintained in strict accordance with the recommendations in the Guide for the Care and Use of Laboratory Animals of the National Institutes of Health using IACUC protocols #19778 (white) and #20968 (green) approved by the UC Davis Institutional Animal Care and Use Committee. Individual sturgeon were euthanized prior to sampling using buffered tricaine methane sulfonate.

## Results

**Fig 1:**
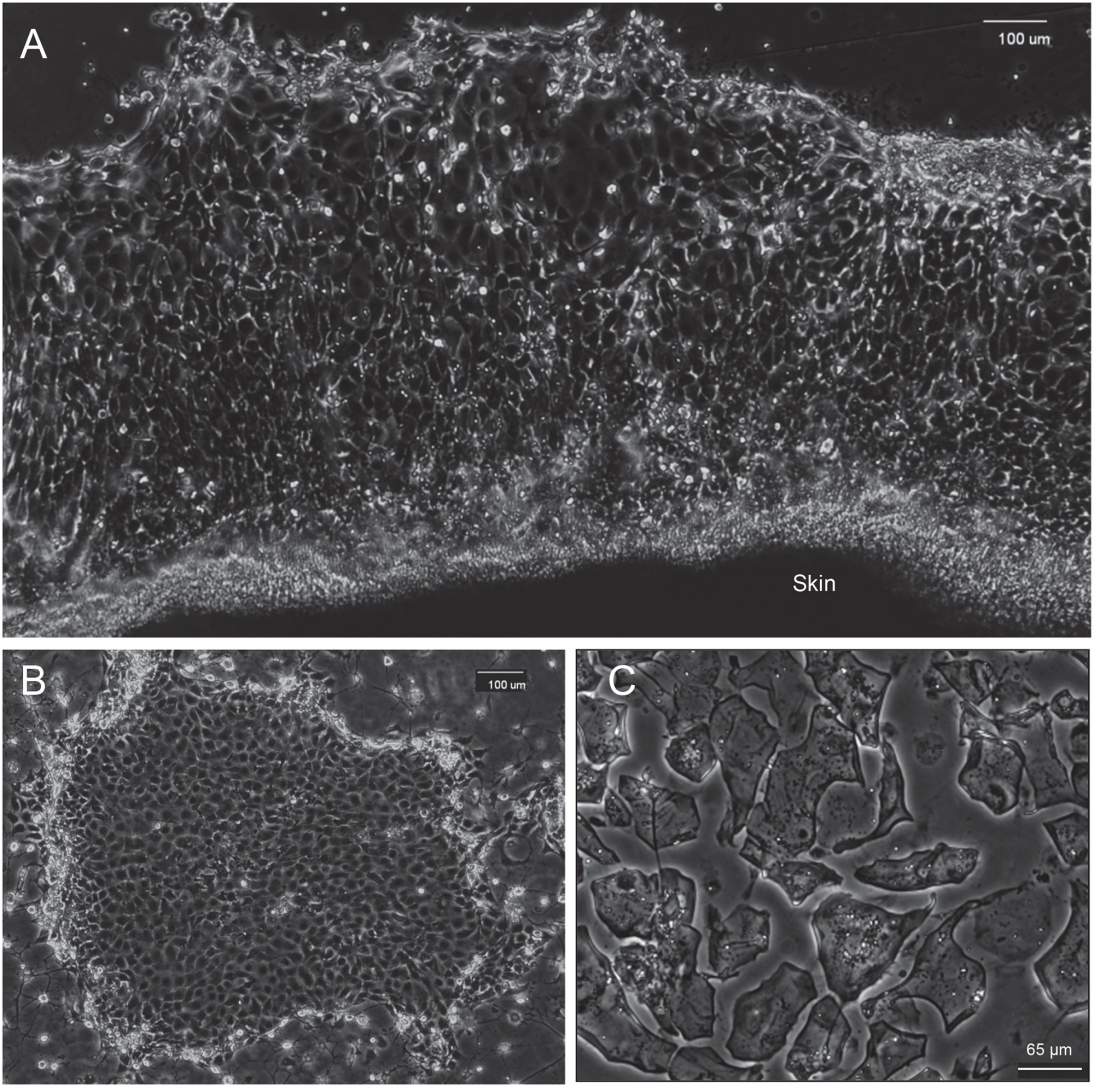
Cultures of sturgeon epidermal cells. (A) Sheet of epithelial cells expanding outward from explant of white sturgeon skin (at bottom of panel). (B) Colony of green sturgeon epidermal cells in primary culture surrounded by sparse 3T3 feeder layer cells. (C) Squame-like cells remaining attached to dish after treatment of confluent culture of white sturgeon cells with ionophore X537A overnight followed by SDS + DTT.

Over the course of two weeks, epithelial cells grew outward as sheets from explanted samples of white sturgeon skin (Fig 1A). In the case of green sturgeon, trypsinizing the skin samples was much more effective. The yield increased with the number of trypsinizations (45 min each), and culturing the remaining tissue after the third round recovered numerous colonies as well. In most cases, the colonies appeared to result from attachment of small clumps of cells (Fig 1B). With both species, the yield of outgrowths or colonies was highest from samples of skin around the protruding mouth. Cell growth was quite slow for the first several passages and gradually increased along with higher colony forming efficiency. By passage 12, newly confluent white sturgeon cultures with a 1:4 split reached confluence again in less than a week. When the medium of highly confluent cultures was replaced by 0.5 mM EDTA in phosphate buffered saline, the cell borders appeared distinctly separate from those of neighboring cells with no clear indication of stratification. Stratification also was not evident microscopically during trypsinization.

**Fig 2:**
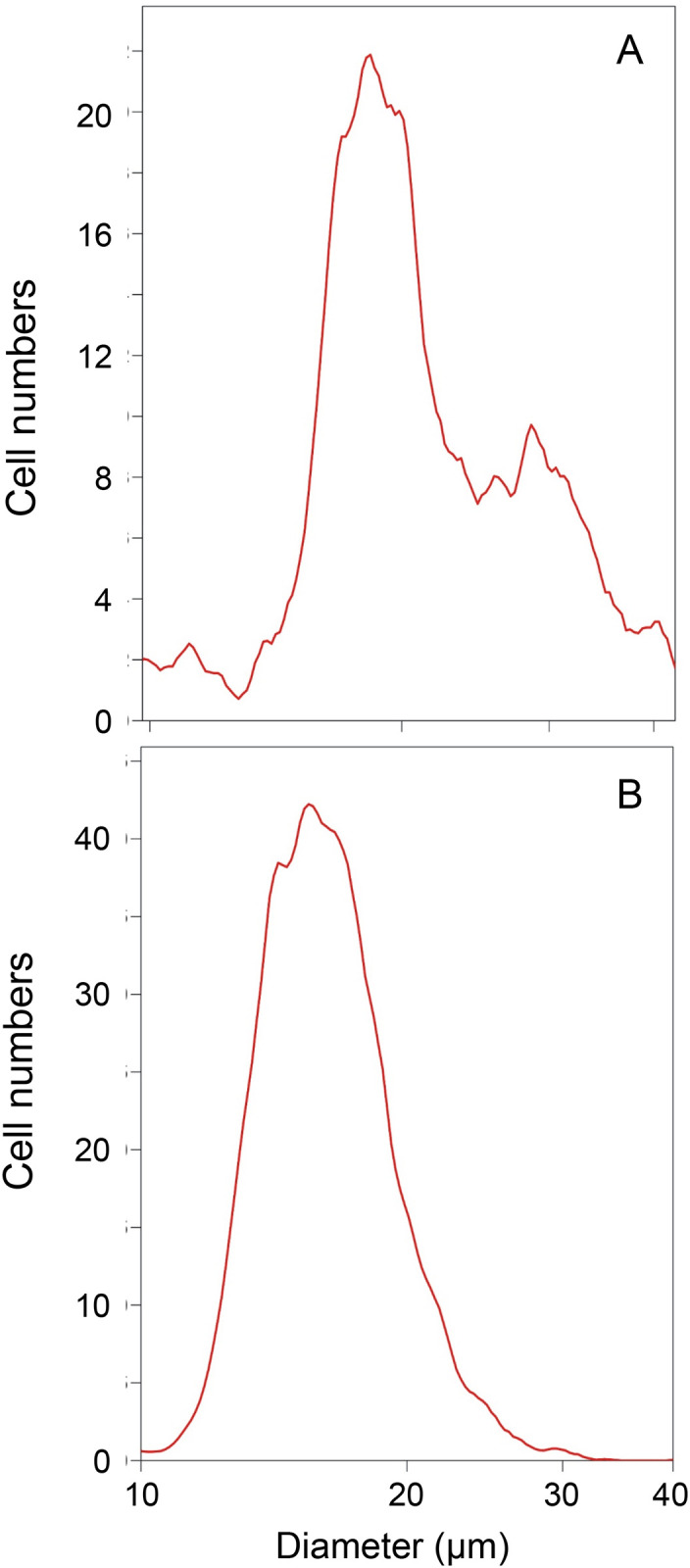
Cell size distribution. (A) Passage 3 white sturgeon epidermal cells confluent for a week and (B) confluent human dermal fibroblasts were trypsinized, suspended in saline and analyzed electronically using a Multisizer 3 Coulter counter adapted for measurement of cell sizes.

When the medium was replaced by a solution of 2% sodium dodecyl sulfate (SDS) and 20 mM DTT, the cells rapidly dissolved. By contrast, cultures that had been pretreated overnight with the ionophore X537A, which raises the cytoplasmic calcium concentration sufficiently to activate transglutaminase activity, did not dissolve. Microscopic observation of cultures to which SDS and DTT were added showed the cells changing in appearance, in many cases losing most internal features, but the majority remained intact (Fig 1C). After detergent treatment, cells that became detached during ionophore treatment were indistinguishable in stability and appearance from those that had remained loosely attached to the dish. Large differences in cell size were noted microscopically and were also observed by measurement of cell size distribution in comparison to human dermal fibroblasts (Fig 2). Such differences were also observed microscopically among the envelopes after ionophore treatment (Fig 1C).

Table 1 shows results of cross-linked envelope formation assays under several conditions. As previously found with cultures from tilapia lip [17], few if any cells (<1%) formed envelopes spontaneously in surface culture. Analogous to the desquamation process observed in human epidermal keratinocyte cultures [37], confluent sturgeon cultures continued to divide slowly despite a lack of space for all the cells to remain attached. When the floating cells were collected four days after the previous medium change, a small but appreciable fraction of them (18% ± 2%) displayed envelopes.

**Table 1:** Cross-linked envelope formation.

Sample	%
Spontaneous*	<1
Desquamated^†^	18 ± 2
Suspended^ǂ^	15 ± 2
X537A^§^	60 ± 5
X537A + IA^§^	1
X537A + Cystamine^§^	≤1

Cells were treated as described below, then counted after subsequent SDS/DTT treatment.

* Confluent cultures treated directly with SDS/DTT.

^†^ Cells shed from confluent cultures and accumulating in the medium over 4 days.

^ǂ^ Trypsinized cells suspended in medium overnight.

^§^ Confluent cultures treated with ionophore X537A (0.1 mg/ml) overnight in the presence or absence of iodoacetamide (25 mM) or 4 hr after addition of cystamine (20 mM).

Trypsin-disaggregated sturgeon cells treated immediately with the ionophore did not form envelopes, unlike human epidermal cell cultures [38]. However, 15% ± 2% of the trypsinized cells formed envelopes in suspension in medium overnight (Table 1), analogous to human epidermal cells [37]. By contrast, the fraction of adherent cells capable of envelope formation upon ionophore treatment was 60% ± 5%, comparable to the degree of envelope formation in cultured human epidermal keratinocytes [38]. To obtain this value, untreated cultures were trypsinized and counted to give total cell numbers, while parallel cultures (without trypsinization) were incubated overnight with ionophore and counted following treatment with SDS and DTT. In such experiments, envelope formation is prevented by treatment of human cells with iodoacetamide or cystamine, which block the active site cysteine of transglutaminase [39, 40] and thereby prevent protein cross-linking (Rice and Green, 1979). Similarly, treating the sturgeon cultures with iodoacetamide or cystamine prevented ionophore induction of envelope formation. As seen in S1 Fig in S1 File, ionophore-inducible envelope formation appeared largely independent of cell density, a feature that distinguishes among epithelial cell lines derived from various rat tissues [41, 42].

Finding that the sturgeon cells were capable of cross-linked envelope formation stimulated a search for expression of a TGM1-like gene in them. Sequencing of the 120 chromosome sterlet sturgeon genome has revealed two TGM1-like genes [33], and it was unknown how many TGM1-like genes would be present in the 240 chromosome white sturgeon with four times the diploid chromosome number [30]. Using those exon sequences to interrogate the white sturgeon genomic DNA revealed two homologous genes, although exons 1 and 2 were not retrieved. (Sequences of a Factor XIII-like and a TGM2-like, but no other TGM1-like, genes were identified.) Using RNA isolated from the cultures permitted cloning these two genes to complete the translated sequences (given in S3 Table in S1 File). As seen in Table 2, the white and sterlet A forms had high levels of amino acid sequence identity (98%) as did the B forms (95%), whereas comparison of A and B forms from each species revealed lower degrees of identity (70–77%). Considerably lower degrees of identity (55–62%) were evident in comparisons of white or sterlet sturgeon Tgm1 A and B forms with those from tilapia, a representative teleost.

**Table 2:** Percent identities in TGM1 sequences generated from two-way comparisons using NCBI Protein BLAST.

	Sterlet A	Sterlet B	White A	White B	Tilapia A
Sterlet B	78				
White A	98	79			
White B	77	95	77		
Tilapia A	60	57	60	55	
Tilapia B	57	58	62	58	63

White refers to white sturgeon. Sequences employed were those indicated by the accession numbers in Table 3.

**Table 3:** Cysteine clusters in sturgeon (white, green, sterlet) compared to representative teleost, amphibians, reptiles and mammals.

Species	Cysteine cluster region (40 residues)	Accession #
Sterlet A	tkrqesr**C**sawmrrv**C**p**C**v**C**kksaddvtdnsgptatmedd	XP_033853104.2
Green sturgeon A	tkrqesr**C**sgwmrrv**C**p**C**v**C**rksadnvtdnsgptatiedd	This work
White sturgeon A	tkrqesr**C**sgwmrrv**C**p**C**v**C**rksaddltdnsgptatiedd	This work
Sterlet B	lktqesrsrgwmgtvfp**C**v**C**tnsadshdvtdynvppatrt	XP_034770304.1
Green sturgeon B	lktqesrsggwlgtvfp**C**v**C**tnsadshdvtdynvrpatrt	This work
White sturgeon B	lktqesrsggwlgtvfp**C**v**C**tnsadshdvtdynvrpatrt	This work
Tilapia A	nvkkrna**C**qewlrkv**C**p**CCC**pkhddvtdtevtgvdepske	XP_019206699.1
Tilapia B	kkqeegg**C**lwwlrkm**C**p**CCC**khpnatsyditdkvetsydi	XP_003456225.1
Cayenne caecilian	pslapsrkkswfqr**CC**g**CC**ssahseedveewrstapgvrd	XP_030042797.1
West. clawed frog	mar**C**eerkksfwerl**C**p**CCC**tersqyepdndmrpvnrpdg	XP_002939073.2
Corn snake	epaprrkkqswfhkf**C**r**CC**aghrddsdwtpapgevpgarr	XP_034261313.1
Sand lizard	etgprrkkrnwfnk**CC**a**CC**sgqgdddwgpapgevpgarre	XP_033026209.1
Green sea turtle	etqperrkrsffskf**C**k**CC**k**CC**agprddtdwgpapgevpg	XP_037771629.1
Chinese alligator	parperrrrgvfskv**C**a**CC**r**CC**agrnddadwgpapgevpg	XP_006039042.2
Swainson’s thrush	sggarglwrrlarg**CC**g**CC**g**CC**gnrdrnrdwepipgevpg	XP_032940238.1
Tasmanian devil	dtrsrgsgrsfwar**CC**s**CC**s**C**rggadddwgpepagprgsg	XP_003755945.1
Human	grsrrgggrsfwar**CC**g**CC**s**C**rnaadddwgpepsdsrgrg	NP_000350.1

Segments of 40 residues are given centered on the cysteine cluster. Cysteine residues are capitalized and in bold.

Among TGM1s, the least identity is observed at the amino terminal end of the protein, probably due to its encoding in an exon added after expansion of the gene family [43]. However, it contains a cluster of cysteine residues that are palmitoylated, permitting membrane attachment. As shown in Table 3, the cluster encoded by the sturgeon genome has the form CPCVC in TGM1A, which differs by one C residue from the typical cluster in teleosts of CPCCC [17], versus FPCVC in TGM1B. Studies of human TGM1 indicate that two strategically placed cysteines are sufficient for membrane anchorage and indicate that hydrophobic residues within the cluster assist in membrane anchorage [44].

**Fig 3:**
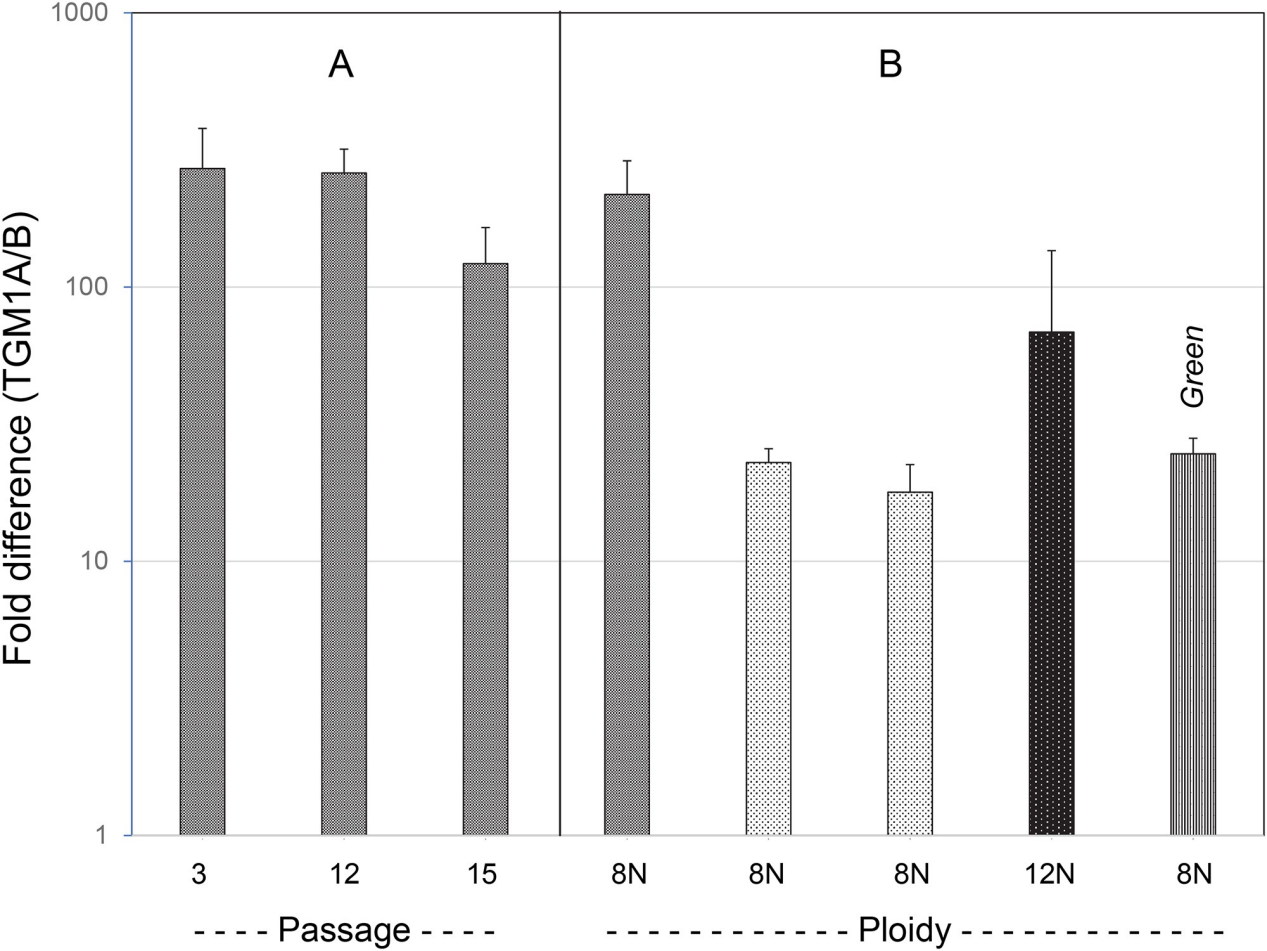
Relative expression levels of TGM1 A and B determined by real time PCR. (A) RNA was isolated from the indicated passages of cells from a single sturgeon. (B) The average ratio TGM1A/TGM1B in panel A is shown along with the ratios for two other octoploid (8N) white sturgeon (lighter shading), a dodecaploid white sturgeon (12N, dark shading) and a green sturgeon (as labeled). Error bars indicate values derived from 2 or 3 independent cultures except for P3 (7 cultures).

TGM1A expression levels were considerably higher than those of TGM1B as judged by real time PCR of mRNA from the cultured cells. The ratio of TGM1A to TGM1B was ≈200 in cells cultured from one white sturgeon, a value that appeared largely independent of the passage number of the cells. In contrast, the ratio was ≈20 in cells cultured from two other octoploid white sturgeon and from one green sturgeon (Fig 3). The ratio observed in cultures from the dodecaploid white sturgeon was intermediate (≈70), indicating the degree of ploidy was not a major determinant.

**Fig 4:**
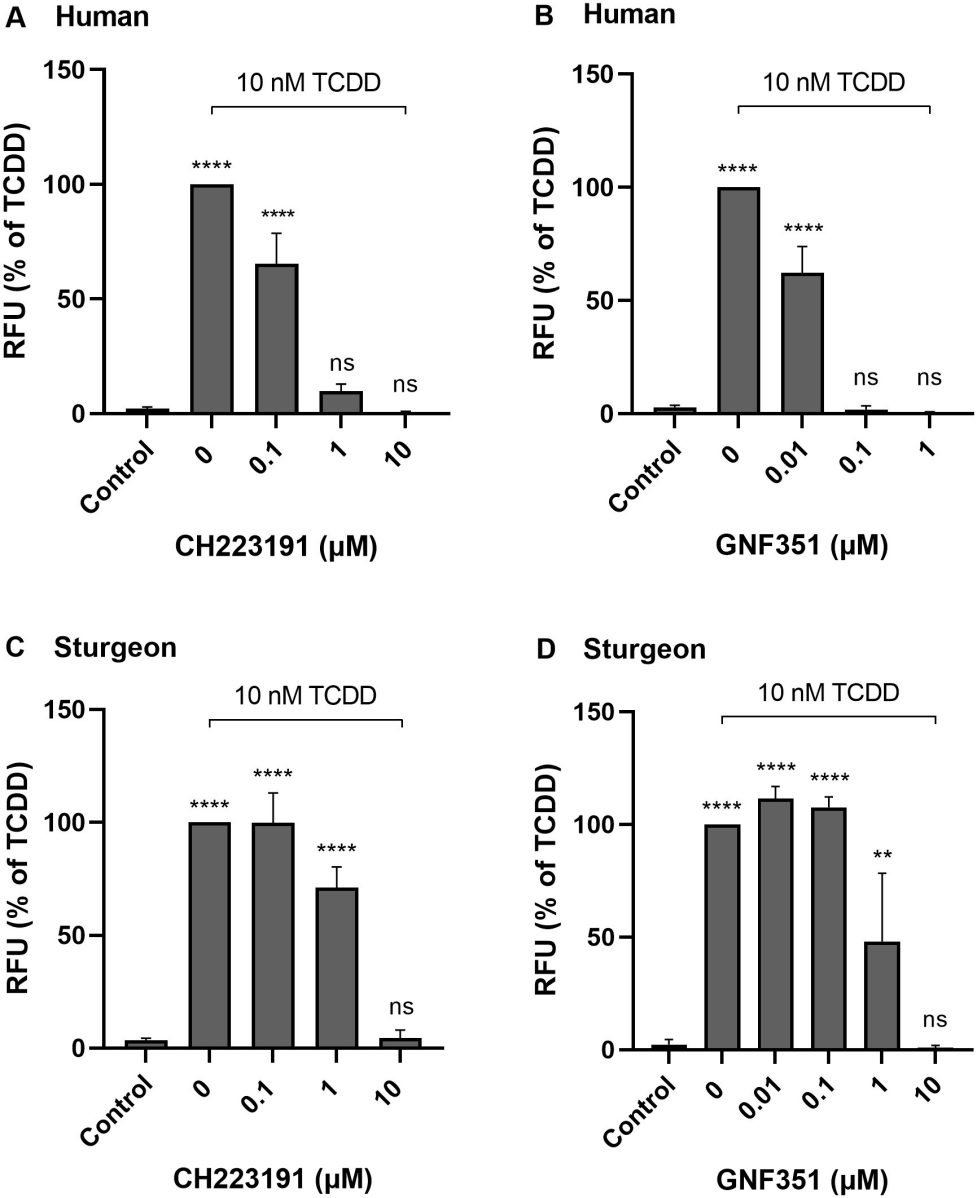
Human and sturgeon epidermal cell responses to TCDD. Human (A, B) and sturgeon (C, D) epidermal cells were pre-treated with indicated concentrations of AHR inhibitors (CH223191 or GNF351) 1 hr before TCDD exposure. After overnight incubation, the cells were treated with 7-ethoxyresorufin in serum free medium for 2 hr and the medium was harvested for EROD assay performed as described in the Methods. The CYP1A activity was corrected for background activity measured from fresh medium containing 7-ethoxyresorufin and expressed as a percent of the CYP1A activity obtained from cells treated with TCDD (set to 100%). Results are presented as the mean ± SD of three independent experiments. Significant differences from untreated control were calculated (one-way ANOVA and Dunnett’s post hoc test) with GraphPad Prism 9 (ns, not significant different; **, p < 0.01; ****, p < 0.0001).

TCDD is the most potent congener of halogenated polycyclic aromatic hydrocarbons, a group of ubiquitous environmental pollutants generated during combustion of organic compounds. TCDD is, therefore, used by the USEPA, World Health Organization and other agencies as an index chemical for exposures and toxicity testing [45]. In this study, we used TCDD to examine the responses of sturgeon epidermal cells to a key class of environmental pollutant. The toxicity of TCDD and other such halogenated polycyclic aromatics is highly dependent on induction of the aryl hydrocarbon receptor signaling pathway, which subsequently induces the expression of a series of related genes such as cytochrome P4501A (CYP1A) [8]. The induction of the xenobiotic-metabolizing enzyme CYP1A can be measured by the EROD assay to monitor the exposure to substances that activate the AHR [46]. As shown in Fig 4, like human keratinocytes, sturgeon cells did respond to TCDD exposure with induced CYP1A activity. Pretreatment with different concentrations of two receptor antagonists, CH223191 and GNF351, inhibited EROD activity in a concentration-dependent manner. Yet, compared to human cells, sturgeon cells required high concentrations of AHR antagonists to produce their inhibitory effects on CYP1A induction, providing another example of species-level differences in AHR ligand sensitivity [47].

## Discussion

TCDD induction of CYP1A in the cultured sturgeon epidermal cells reveals a functioning AHR signaling system as found in many mammalian and fish epithelial cells. The degree of CYP1A induction by TCDD in EROD assays was ≈40 fold higher than in untreated cultures, similar to the induction in white sturgeon liver, intestine, and gill by treatment with the receptor ligand β-naphthoflavone [48]. The cultures thus provide a quantitative alternative to observing CYP1A induction in systems such as zebrafish skin, where it is an effective immunohistochemical biomarker for AHR2 activation [49]. This feature is important for modeling the cellular response to various pollutants that produce deleterious effects directly by receptor activation or indirectly as a result of their biotransformation by CYP1A to electrophiles forming macromolecular adducts. Assaying fluorescence from cleavage of the substrate 7-ethoxy-resorufin is easily adaptable to multiwell plates [50], which may be assisted by the streamlined use of live cells as described here. The contrast in effectiveness in the sturgeon and mammalian cells of two potent inhibitors of AHR2 activation emphasizes that species generally develop differing sensitivities to environmental exposures such as to pollutants. Differences in amino acid sequence in the AHR2 ligand binding domain are evident even among sturgeon species and are anticipated to be manifest as TCDD sensitivity differences [51].

The ability of the sturgeon cells to become greatly enlarged resembles squame formation in human epidermis and in keratinocyte cultures [36], where the size in the latter is influenced by the culture microenvironment [52, 53]. Mechanisms regulating cell size in multicellular organisms are not well understood [54]. While sizes increase in general in preparation for mitosis, the dramatic enlargement keratinocytes undergo is a critical feature of terminal differentiation with functional consequences in this cell type. Further work may help elucidate to what degree the enlargement in these fish cells was repurposed during evolution of terrestrial epidermis.

The capability of epithelial cells derived from the skin of sturgeon to form cross-linked envelopes resembled that from tilapia in several ways. First, treatment of the cultured cells with an ionophore permitting cytoplasmic calcium ion influx induced formation of envelope-like structures visible microscopically in a majority of the cells after detergent treatment. Second the envelopes did not form spontaneously but required treatment, in this case by the ionophore. Third, envelope-forming capability could be attributed to a calcium-requiring keratinocyte transglutaminase, a TGM1-like enzyme. In contrast to cultured human keratinocytes, however, this ability to form envelopes was not stimulated rapidly by ionophore immediately after trypsinization. This observation may reflect more substantial preparation for terminal differentiation in human keratinocytes such as expression of proteins not present in fish [55] or mobilization of acylceramides for fabrication of the lipid barrier [24], either stabilizing the cell periphery.

The data in Table 3 indicate that sturgeon speciation occurred much later than the genome duplication leading to TGM1 divergence into A and B forms. Sterlet and white sturgeons diverged from a common lineage approximately 120 million years ago judging by mitochondrial cytochrome b sequences [56]. The level of amino acid identity of the TGM1A forms between sterlet and white sturgeons, or the degree of identity of TGM1B forms between these two species (95–98%), was considerably higher than the identity when A and B forms were compared within or between species (77–79%). An approximately linear rate of amino acid substitution over time is consistent with the duplication occurring prior to the divergence of cartilaginous sturgeon and bony teleost lineages some 400 million years ago. Whether the agnathans have A and B forms remains to be seen. Although the molecular cloning in present work was sufficient to distinguish A and B forms, expression of closely related forms of either type from homologous sequences on the polyploid chromosomes cannot be ruled out. By contrast, four TGM1 sequences were distinguishable in sequences of Atlantic salmon (*Salmo salar*) arising from two genome duplications [17]. Comparison of the two TGM1A or the two TGM1B forms showed higher levels of identity (84–87%) than comparison between the A and B forms (70–73%).

Analogous to expression in tilapia, where TGM1A and TGM1B were detected in the stratified lip and oral epithelia [17], the TGM1 enzymes were expressed in cells derived from sturgeon epidermis. Although cells with intermediate filaments have been reported in sturgeon epidermis [28], more detailed characterization awaits, including whether these or other cells express TGM1-like enzymes in the epidermis *in vivo*. The observed high fraction of envelope-forming cells in culture could reflect abundant expression *in vivo*, or it could result from selection for a minority of keratinocyte-like cells. A third possibility is that induction of TGM1-like gene expression is part of a re-differentiation process in the cultured cells that migrate outward from tissue explants. A possibly analogous phenomenon occurs during migration of mouse cranial neural crest cells that develop pluripotent character [57]. More specifically, numerous epithelia in the rat undergo reversible squamous metaplasia as a result of vitamin A deprivation [58], a reprogramming process evident in cell culture resulting in TGM1 induction and ionophore-inducible envelope formation in this species [42, 59].

A lack of TGM1, and thus a defective cross-linked protein envelope, is a major cause of the human scaly skin disease lamellar ichthyosis [60, 61], and mice with the gene ablated are not viable after birth [62]. We speculate this epidermal function developed from repurposing a TGM1-like enzyme in agnathans necessary to form their isopeptide cross-linked teeth [29] and in sturgeon to form the keratinized spines in the skin [23]. Envelope formation occurs during terminal differentiation in mammalian epidermis and is observed spontaneously in their cultured keratinocytes, albeit at a low level under optimal growth conditions. To permit proper barrier function in a terrestrial environment, a TGM1 activation process for protein cross-linking at the granular layer of mammalian epidermis appears necessary. Evolution of the trigger for cross-linking, satisfactory substrate proteins for envelope formation, and the subsequent process of lipid attachment all remain to be elucidated.

## Supporting information

S1 File (PDF)

## References

[1] Hildebrand LR, Drauch Schreier A, Lepla K, McAdam SO, McLellan J, Parsley MJ, et al. Status of white sturgeon (Acipenser transmontanus Richardson, 1863) throughout the species range, threats to survival, and prognosis for the future. J Appl Ichthyol. 2016;(Suppl 1):261–312. doi: 10.1111/jai.13243

[2] Moser ML, Israel JA, Neuman M, Lindley ST, Erickson DL, McCovey BWJ, et al. Biology and life history of green sturgeon (Acipenser medirostris Ayres, 1854): state of the science. J Appl Ichthyol. 2016;32 Suppl 1:67–86. doi: 10.1111/jai.13238

[3] Adams PB, Grimes C, Hightower JE, Lindley ST, Moser ML, Parsley MJ. Population status of North American green sturgeon, Acipenser medirostris. Environ Biol Fish. 2007;79:339–56. doi: 10.1007/s10641-006-9062-z

[4] Pikitch EK, Doukakis P, Lauck L, Chakrabarty P, Erickson DL. Status, trends, and management of sturgeon and paddlefish fisheries. Fish and Fisheries. 2005;6:233–65. doi: 10.1111/j.1467-297.2005.00190.x

[5] Moyle PB, Quiñones RM, Katz JV, Weaver J. Fish species of special concern in California. California Department of Fish and Wildlife. Sacramento 2015.

[6] Gilbreath AN, McKee LJ. Concentrations and loads of PCBs, dioxins, PAHs, PBDEs, OC pesticides and pyrethroids during storm and low flow conditions in a small urban semi-arid watershed. Sci Total Environ. 2015;526:251–61. doi: 10.1016/j.scitotenv.2015.04.052 25955693

[7] Overdahl KE, Sutton R, Sun J, DeStefano NJ, Getzinger GJ, Ferguson PL. Assessment of emerging polar organic pollutants linked to contaminant pathways within an urban estuary using non-targeted analysis. Environ Sci Process Impacts. 2021;23:429. doi: 10.1039/d0em00463d 33656498 PMC9136708

[8] King-Heiden TC, Mehta V, Xiong KM, Lanham KA, Antkiewicz DS, Ganser A, et al. Reproductive and developmental toxicity of dioxin in fish. Molec Cell Endocrinol. 2012;354:121–38. doi: 10.1016/j.mce.2011.09.027 21958697 PMC3306500

[9] Shankar P, Dasgupta S, Hahn ME, Tanguay RL. A review of the functional roles of the zebrafish aryl hydrocarbon receptors. Toxicol Sci. 2020;178:215–38. doi: 10.1093/toxsci/kfaa143 32976604 PMC7706399

[10] Souder JP, Gorelick DA. ahr2, but not ahr1a or ahr1b, is required for craniofacial and fin development and TCDD dependent cardiotoxicity in zebrafish. Toxicol Sci. 2019;170:25–44. doi: 10.1093/toxsci/kfz075 30907958 PMC6592186

[11] Palumbo AJ, Denison MS, Doroshov SI, Tjeerdema RS. Reduction of vitellogenin synthesis by an aryl hydrocarbon receptor agonist in the white sturgeon (Acipenser transmontamus). Environ Toxicol Chem. 2009;28:1749–55. doi: 10.1897/08-481.1 19292566 PMC2858920

[12] Gardell AM, Qin Q, Rice RH, Li J, Kültz D. Derivation and osmotolerance characterization of three immortalized tilapia (*Oreochromis mossambicus*) cell lines. PLoS One. 2014;9(5):e95919. doi: 10.1371/journal.pone.0095919 24797371 PMC4010420

[13] Todaro GJ, Green H. Quantitative studies of the growth of mouse embryo cells in culture and their development into established lines. J Cell Biol. 1963;17:299–313. doi: 10.1083/jcb.17.2.299 13985244 PMC2106200

[14] Rheinwald JG, Green H. Formation of a keratinizing epithelium in culture by a cloned cell line derived from a teratoma. Cell. 1975;6:317–30. doi: 10.1016/0092-8674(75)90183-x 1052770

[15] Allen-Hoffmann BL, Rheinwald JG. Polycyclic aromatic hydrocarbon mutagenesis of human epidermal keratinocytes in culture. Proc Natl Acad Sci USA. 1984;81:7802–6. doi: 10.1073/pnas.81.24.7802 6440145 PMC392240

[16] Chapman S, Liu X, Meyers C, Schlegel R, McBride AA. Human keratinocytes are efficiently immortalized by a Rho kinase inhibitor. J Clin Invest. 2010;120:2619–26. doi: 10.1172/JCI42297 20516646 PMC2898606

[17] Rodriguez Cruz SI, Phillips MA, Kültz D, Rice RH. Tgm1-like transglutaminases in Tilapia (Oreochromis mossambicus). PLoS One. 2017;12(5):e0177016. doi: 10.1371/journal.pone.0177016 28472103 PMC5417640

[18] Bi X, Wang K, Yang L, Pan H, Jiang H, Wei Q, et al. Tracing the genetic footprints of vertebrate landing in non-teleost ray-finned fishes. Cell. 2021;184:1–15. doi: 10.1016/j.cell.2020.12.019 33545088

[19] Meyer A, Schloissnig S, Franchini P, Du K, Woltering JM, Irisarri I, et al. Giant lungfish genome elucidates the conquest of land by vertebrates. Nature. 2021;590:284–9. doi: 10.1038/s41586-021-03198-8 33461212 PMC7875771

[20] Wang K, Wang J, Zhu C, Yang L, Ren Y, Ruan J, et al. African lungfish genome sheds light on the vertebrate water-to-land transition. Cell. 2021;184:1362–76. doi: 10.1016/j.cell.2021.01.047 33545087

[21] Hawkins MB, Henke K, Harris MP. Latent developmental potential to form limb-like skeletal structures in zebrafish. Cell. 2021;184:899–911. doi: 10.1016/j.cell.2021.01.003 33545089

[22] Dong Y-W, Blanchard TS, Noll A, Vasquez P, Schmitz J, Kelly SP, et al. Genomic and physiological mechanisms underlying skin plasticity during water to air transition in an amphibious fish. J Exp Biol. 2021;224:jeb235515. doi: 10.1242/jeb.235515 33328287 PMC7860121

[23] Yang S, Fu HM, Xiao Q, Liu Q, Wang Y, Yan TM, et al. The structure of the skin, types and distribution of mucous cell of Yangtze sturgeon (Acipenser dabryanus). Int J Morphol. 2019;37:541–7. doi: 10.4067/S0717-95022019000200541

[24] Akiyama M. Corneocyte lipid envelope (CLE), the key structure for skin barrier function and ichthyosis pathogenesis. J Dermatol Sci. 2017;88:3–9. doi: 10.1016/j.jdermsci.2017.06.002 28623042

[25] Karim N, Phinney BS, Salemi M, Wu P-W, Naeem M, Rice RH. Human stratum corneum proteomics revreals cross-linking of a broad spectrum of proteins in cornified envelopes. Exp Dermatol. 2019;28:618–22. doi: 10.1111/exd.13925 30916809

[26] Feingold KR, Elias PM. Role of lipids in the formation and maintenance of the cutaneous permeability barrier. Biochim Biophys Acta. 2014;1841:280–94. doi: 10.1016/j.bbalip.2013.11.007 24262790

[27] Wiley ML, Collette BB. Breeding tubercles and contact organs in fishes: their occurrence, structure and significance. Am Mus Nat Hist. 1970;143:143–216.

[28] Shute L, Huebner E, Anderson WG. Microscopic identification of novel cell types in the integument of larval lake sturgeon, Acipenser fulvescens. J Morphol. 2016;277:86–95. doi: 10.1002/jmor.20480 26440535

[29] Rice RH, Wong VJ, Pinkerton KE. Ultrastructural visualization of cross-linked protein features in epidermal appendages. J Cell Sci. 1994;107:1985–92. doi: 10.1242/jcs.107.7.1985 7983163

[30] Gille DA, Famula TR, May BP, Schereier AD. Evidence for a maternal origin of spontaneous autopolyploidy in culturedwhite sturgeon (Acipenser transmontanus). Aquaculture. 2015;435:467–74. doi: 10.1016/j.aquaculture.2014.10.002

[31] Rice RH. Assays for involucrin, transglutaminase and ionophore-inducible envelopes. In: Leigh FMW I. M., Lane B., editor. Keratinocyte Methods. UK: Cambridge University Press; 1994. p. 157–65.

[32] Weisenfeld NI, Kumar V, Shah P, Church DM, Jaffe DB. Direct determination of diploid genome sequences. Genome Res. 2017;27:757–67. doi: 10.1101/gr.214874.116 28381613 PMC5411770

[33] Du K, Stöck M, Kneitz S, Klopp C, Woltering JM, Adolfi MC, et al. The sterlet sturgeon genome sequence and the mechanisms of segmental rediploidization. Nat Ecol Evol. 2020;4:841–52. doi: 10.1038/s41559-020-1166-x 32231327 PMC7269910

[34] Coomes MW, Sparks RW, Fouts JR. Oxidation of 7-ethoxycoumarin and conjugation of umbelliferone by intact, viable epidermal cells from the hairless mouse. J Invest Dermatol. 1984;82:598–601. doi: 10.1111/1523-1747.ep12261390 6725983

[35] Heimann R, Rice RH. Polycyclic aromatic hydrocarbon toxicity and induction of metabolism in cultivated esophageal and epidermal keratinocytes. Cancer Res. 1983;43:4856–62. 6192910

[36] Sun T-T, Green H. Differentiation of the epidermal keratinocyte in cell culture: Formation of the cornified envelope. Cell. 1976;9:511–21. doi: 10.1016/0092-8674(76)90033-7 1009573

[37] Green H. Terminal differentiation of human epidermal cells. Cell. 1977;11:405–15. doi: 10.1016/0092-8674(77)90058-7 302145

[38] Rice RH, Green H. Presence in human epidermal cells of a soluble protein precursor of the cross-linked envelope: Activation of the cross-linking by calcium ions. Cell. 1979;18:681–94. doi: 10.1016/0092-8674(79)90123-5 42494

[39] Folk JE, Chung SI. Molecular and catalytic properties of transglutaminases. Advances in enzymology and related areas of molecular biology. 1973;38:109–91. doi: 10.1002/9780470122839.ch3 4151471

[40] Siefring GEJ, Apostol AB, Velasco PT, Lorand L. Enzymatic basis for the Ca++-induced cross-linking of membrane proteins in intact human erythrocytes. Biochemistry. 1978;17:2598–604. doi: 10.1021/bi00606a022 28146

[41] Heimann R, Rice RH. Rat esophageal and epidermal keratinocytes: Intrinsic differences in culture and derivation of continuous lines. J Cell Physiol. 1983;117:362–7. doi: 10.1002/jcp.1041170311 6197421

[42] Phillips MA, Rice RH. Convergent differentiation in cultured rat cells from nonkeratinized epithelia: Keratinocyte character and intrinsic differences. J Cell Biol. 1983;97:686–91. doi: 10.1083/jcb.97.3.686 6193127 PMC2112584

[43] Phillips MA, Stewart BE, Rice RH. Genomic structure of keratinocyte transglutaminase. Recruitment of new exon for modified function. J Biol Chem. 1992;267:2282–6. 1346394

[44] Phillips MA, Qin Q, Mehrpouyan M, Rice RH. Keratinocyte transglutaminase membrane anchorage: Analysis of site-directed mutants. Biochemistry. 1993;32:11057–63. doi: 10.1021/bi00092a015 8105889

[45] Van den Berg M, Birnbaum LS, Denison M, De Vito M, Farland W, Feeley M, et al. The 2005 World Health Organization reevaluation of human and Mammalian toxic equivalency factors for dioxins and dioxin-like compounds. Toxicol Sci. 2006;93:223–41. doi: 10.1093/toxsci/kfl055 16829543 PMC2290740

[46] Petrulis JR, Chen G, Benn S, LaMarre J, Bunce NJ. Application of the ethoxyresorufin-O-deethylase (EROD) assay to mixtures of halogenated aromatic compounds. Environ Toxicol. 2001;16:177–84. doi: 10.1002/tox.1022 11339718

[47] Denison MS, Soshilov AA, He G, DeGroot DE, Zhao B. Exactly the same but different: promiscuity and diversity in the molecular mechanisms of action of the aryl hydrocarbon (dioxin) receptor. Toxicol Sci. 2011;124:1–22. 093/toxsci/kfr218 doi: 10.1093/toxsci/kfr218 21908767 PMC3196658

[48] Doering JA, Wiseman S, Beitel SC, Tendler BJ, Giesy JP, Hecker M. Tissue specificity of aryl hydrocarbon receptor (AhR) mediated responses and relative sensitivity of white sturgeon (Acipenser transmontanus) to an AhR agonist. Aquat Toxicol. 2012;114–115:125–33. doi: 10.1016/j.aquatox.2012.02.015 22446824

[49] Shankar P, Geier MC, Truong L, McClure RS, Pande P, Waters KM, et al. Coupling genome-wide transcriptomics and developmental toxicity profiles in zebrafish to characterize polycyclic aromatic hydrocarbon (PAH) hazard. International journal of molecular sciences. 2019;20:2570. doi: 10.3390/ijms20102570 31130617 PMC6566387

[50] Schiwy A, Brinkmann M, Thiem I, Guder G, Winkens K, Eichbaum K, et al. Determination of the CYP1A-inducing potential of single substances, mixtures and extracts of samples in the micro-EROD assay with H4IIE cells. Nature Protocols. 2015;10:1728–41. doi: 10.1038/nprot.2015.108 26448361

[51] Doering JA, Farmahin R, Wiseman S, Beitel SC, Kennedy SW, Giesy JP, et al. Differences in activation of aryl hydrocarbon receptors of white sturgeon relative to lake sturgeon are predicted by identities of key amino acids in the ligand binding domain. Environ Sci Technol. 2015;49:4681–9. doi: 10.1021/acs.est.5b00085 25761200

[52] Ngo MA, Sinitsyna NN, Qin Q, Rice RH. Oxygen dependent differentiation of human keratinocytes. J Invest Dermatol. 2007;126:2507–15. doi: 10.1038/sj.jid.5700522 16977326

[53] Reznikova TV, Phillips MA, Patterson TJ, Rice RH. Opposing actions of insulin and arsenite converge on PKCδ to alter keratinocyte proliferative potential and differentiation. Molec Carcinogen. 2010;49:398–409. doi: 10.1002/mc.20612 20082316 PMC3152260

[54] D’Ario M, Sablowski R. Cell size control in plants. Ann Rev Genet. 2019;53:45–65. doi: 10.1146/annurev-genet-112618-043602 31430180

[55] Strasser B, Mlitz V, Hermann M, Rice RH, Eigenheer RA, Alibardi L, et al. Evolutionary origin and diversification of epidermal barrier proteins in amniotes. Molec Biol Evol. 2014;31:3194–205. doi: 10.1093/molbev/msu251 25169930 PMC4245816

[56] Peng Z, Ludwig A, Wang D, Diogo R, Wei Q, He S. Age and biogeography of major clades in sturgeons and paddlefishes (Pisces: Acipenseriformes). Molecular Phylogenetics and Evolution. 2007;42:854–62. doi: 10.1016/j.ympev.2006.09.008 17158071

[57] Zalc A, Sinha R, Gulati GS, Wesche DJ, Daszczuk P, Swigut T, et al. Reactivation of the pluripotency program precedes formation of the cranial neural crest. Science. 2021;371:586. doi: 10.1126/science.abb4776 33542111 PMC8557957

[58] Wolbach SB, Howe PR. Tissue changes following deprivation of fatsoluble A vitamin. J Exp Med. 1925;42:753–78. doi: 10.1084/jem.42.6.753 19869087 PMC2131078

[59] Parenteau NL, Pilato A, Rice RH. Induction of keratinocyte type-I transglutaminase in epithelial cells of the rat. Differentiation. 1986;33:130–41. doi: 10.1111/j.1432-0436.1986.tb00418.x 2436965

[60] Huber M, Rettler I, Bernasconi K, Frenk E, Lavrijsen SPM, Ponec M, et al. Mutations of keratinocyte transglutaminase in lamellar ichthyosis. Science. 1995;267:525–8. doi: 10.1126/science.7824952 7824952

[61] Russell LJ, DiGiovanna JJ, Rogers GR, Steinert PM, Hashem N, Compton JG, et al. Mutations in the gene for transglutaminase 1 in autosomal recessive lamellar ichthyosis. Nat Genet. 1995;9:279–83. doi: 10.1038/ng0395-279 7773290

[62] Matsuki M, Yamashita F, Ishida-Yamamoto A, Yamada K, Kinoshita C, Fushiki S, et al. Defective stratum corneum and early neonatal death in mice lacking the gene for transglutaminase 1 (keratinocyte transglutaminase). Proc Natl Acad Sci USA. 1998;95:1044–9. doi: 10.1073/pnas.95.3.1044 9448282 PMC18665

