# Supplementary material for: Epidermal cell cultures from white and green sturgeon (Acipenser transmontanus and medirostris): Expression of TGM1-like transglutaminases and CYP4501A
Source: PLoS One. 2022 Mar 16;17(3):e0265218. doi: 10.1371/journal.pone.0265218 (PMC8926185; doi:10.1371/journal.pone.0265218)
Supplement: S1 File — (PDF) [file pone.0265218.s001.pdf]

# Supporting Information

# Sturgeon Epidermal Cell Culture

**S1 Table. Genomic sequences obtained from white sturgeon unfinished genomic DNA sequence by tBLASTn using sterlet sturgeon TGM1A and TGM1B amino acid sequences.** Coding regions (exons) are highlighted in red. Interrogation of the unfinished genomic sequence with amino acid sequences corresponding to exons 1-2 for sterlet forms A or B did not reveal any good alignments.

**TGM1A sterlet (accession XP_033853104.2) interrogation.** The genomic sequence was obtained as a single continuous alignment for exons 3-14.

AGATCTGATATTATCGGTAAAGAGTGTTGATTTGATTAAATCTCGGTCTGGAATTAACCGGAAGGAGCATCACA
CAGACGAATTTGAATACGATGACCTCATCCTGCGCCGCGGCCAATCGTTCGTCATGGAAATCGAATTCTCGCGA
CCTTTCAACCCCGACAATGACACCATGCACTTGGAACTGCAGATCGGTGAGTCAAGACTGGGAAATGATTGCAA
ACATCAGAACAAGATGAGGGGGCTTGCGTTTAGTCCAAGGTGGCTAGATAGCCTAAATGTCTTTATATTAGGTA
GTGACAGCGCCATCTAGTGCACAGTTAATGTATTGCCAGCAAAACAAACTTGATCCAAAGTGTCAGATCACTAG
ATGCACTGGCTCTTAATTCAATAAAGATACATTTTCTAGCCTACAGATATTTATGACAGGCAGCTGAACTGATG
AGCAGCTCCTGAACTCATAATGAAAACACAATAACACAATATTTCACTATTTGCAATAGAAATATAAGGAGGCA
AAATAAATAAATCTAGCCTGTGTTACACATGTAACAGCTCCTGAACTCACAGTCAAAGCAACACAGACAAAAAT
TAAAACGATTGCAAACATAACAAGCTAAAGGAACAATAAAAAAAGAATAAGAAACTATAAGCTTGAACTCTTTA
AAGAAGTGTCAGTGGGGGCGAGCTGTTACAACTTTGGTAACTGCTCATCACTCCAGTGTACATTTAGAAATACT
GAACTTTGAACTACAACATTGAGAAATACTTCTTGTGGAAACATTGGTCCTCACCTTAAAACTGCCTTTAAATT
GTGGTTTGAGTAGCAAGTTCAGTGATCTTTTGACCCACTTTCAGTTTCTATAAACAGCAAGTCCTTATTTCAGT
TCCCTAACTGAGTTTGAAGACTGTGCCAGCAGGCTGTCTCATGACTGTGTTAATGTCTCAAAGACTACCAGGCA
CTGCATTCACAGAATTGTGCGTTCATCTTGAACCGAGAGCGCAAGACATCCACACAAAAATATCTGCACTGCAT
TATAGCACCAGTGTTACTTGATAACTACTTATCCCCTGCTGGTAATGAAAGCCCTTGTGGATACAGTCGTAAGC
ACGCATGTCACACTGCACACATATTATTATTTTTTATTATTATTTATTTCTTAGCAGACGCCCTTATCCAGGGC
AACTTACAATTGTTACAAGATATCACATTATTTTTACATACAATTACCCATTTATACAGTTGAGTTTTTACTGG
AGCAATCTAGGTAAAGTACCTTGCTCAAGGGTACAACAGCAGTGTCCCCGACCGGGGATTGAACCCATGACCCT
CCGGTCAAGAGTCCACAGCTCTAACCACTACTCCACACTGCTGCCCATATCTACATATCTGGAGAACCACTTAT
CTCATTGCCATGAAATAGACTGAATGGATCCCTCGAGACACCTTCAGACACCTTGTGAGTCTAGACCAGGTTAA
GGCTGTGATCAAGGCAAATGGGGTCCCGACCCGATATTAGGGACAGGTCCTAATAAAGTAATCTAACTTCTTGA
GTTTTAATAACTGTGTTATGTATTGAATATTCACTGTGGAAAGAGAAGAAGGATATGAGTGGGATAGTCAGATT
CAGTTATAGCCTTTTCATACCACGATTGAGAAATTTCCTGCACATGTTTCTGACCTCTCAGCTTAAAACCACAG
GGATGGAAATAAGACTCCTATTGAATAGCAGTTTCACCCAAGTTTTACTACCAGCTTGATTAGGCACAGTGTAT
ATGTAAAAAAACACAGGTGTGTCTTATTAATCTAATAATGCAATTAAGAATGAATCAAACTGTTAATGGGTGTC
GTAGTGCCATCCCCTTAGTTTTTTGATATTTGCAGTCGTTCAGGGAGATGGGGGAGAGGGAGGCGTAGGTGGGG
TAGAAGGGAGAGGGGAGAGGCAGGAAAAGAACAAAGGGAGAGACTCGAGACTGAATGGTAAGATTGAGGTGCCC
AAGACATGTTCACAATATGGGAGCGGTTGTCTGCAGTGTGTGGGTAGCAATGGAAGAGTGGAGGCCTAACTTTA
GCCCAAGGTATCCGGTTGCATCAGTTCAGGCACAAGGTCATGTTTCAAAATGCAGGCTATCTATAGACATTGCT
TATTAGATATACAGACAGCCCCCCTGGGCTTCAATCTGCATAGTTTTATATATTATACAGTAACACTAAACATG
ATTCAGACAGTCCCCCTGAGTTTCAATCTGCATACTATTAGTCACATGCCTTGTGGTTTCCCATTCAAACTGAA
AGCTAGTCATTAGATTGAACCCACACATAAACTTTGAACTCCCAAACGGTTTTAAAAATAGTTTTTCCAACACA
GTATTTCTCAGTTGTGAACTTATAACTAATATAAAACACTATGGGACACTTTTATAAGGGTTAGTTTACCATGT
TTTTTTTTTTTAAGAATAAATTATATTGTAGTGTCAAACTCAACAGGATTGTGTGGCTCTGTCTGATGTCTGAT
GTCTGTCTGTCTGTCTGTCTGTAGCTCTAATGAAACTTAACTCTTTTCAGAATGTAGTAAACTAACAGACATCA
ATAAGAAAGGAATTATATTGTTGTGTCAAACTGAACAGTACAAATACTCAATAGTAATATTCTGACTGTGGGGG
CTGGCATTTTTCAAGAAGATTCTCAAGCAGTTGAAGAGTTAAGTCTGTGAAATTCCTGAGAGGCTTGAACGGGA
AAAATATTTGTCAAGAGAGGCCAGCATACCTGGCACTGAGCTGAGAGAACGAGAGGGGAGACAGCAGCTAGAGA
ATGTTCCAGCAGTGATGCAGAGCTGAGCCCACTTAAGAAATTAACTATACCCTTCACAAATGATTTTCAATTTG
CAATGCAGACAAACAGTGGCAATAATGGTTCTGTATTATACTGAGGGACAATGGTAGCACAAGTATAGGGCAGC

TACAATGGGGTGAGGCTGGTAGAATGGGACCACAGTGTGCTAACTATACCCTAATTGATCTCCAACGGCAATGC
AGATAGACAGTGGCACTGCAATGGCAATAATGGTTCTGTAGCTTCTGAGAAAGGTGAAGGACGAGTGAATGAAT
CTCTGCCTTTGTAATGTAACCTTATATTAATAATAGTAATAATAATAGCAGCAGTTATGCAGTTATTTTTTAAA
AATATTTATTTTTATTTCCAGGTCCACTGCCACAGGTGTCAAAGGGCACCCATGTCATCATTCCATTGGTCGAG
GAGCTGGAAGACAACCGATGGGAAGCCAAGATTGTGCAGAGGGTGGGGACTAAGATGAAACTGTCTGTCGGATC
CCCGGTGACAGCTGTGATTGGCCGGTACAAGTTTGCAGTGGCCACTCACAGCTCGGGGGGAGACTTCAAGATGG
AACACAACCCCAAGAACGACATCACCTTCCTGTTCAACCCCTGGTGCGAAAGTAAGTGTTTTCCCCACTGCAAT
GAGGCTCGGTTTTATTTGGGAGATTGGTTCCTACCTAAAAATCAGAGCAAGAAAAAAAATAAAGAAAAAAGAAA
GATTGAGACTTAGTTTTATAGTCGTGTGTGTGTGTGTGTTTTTTTTCCAGCTTGACCCTCAAAGCCACCTCTCT
TACGCTGAATTATATATCTACTTCTGTTTTTCTAAATAACACACACACACAAAAAAAACTTAAGAACATAAGAA
AGTTTACAAACGAGAGGAGGCCATTCAGCTCATCTTGCTCACTTGGTTGTAGCTTATTGATCCCAGAATCTCAT
CAAGCAGCTTCTTGAAGGATTCCAGGGTGTCAGCTTCAACAACATTACTGGGGAGTTGGTTCCAGACCCTCACA
ATTCTCAGTGTAAAAAAGTGCCTCCTATTTTAAATAGCAACTTGGGTCTGAAATATCCCAGCTATCTTAATGTA
CTCTTCGTGTTCAGATTATGGGGACTTTTTCAAAAGTTTAAAACAAACCCTGTGATCTCTATAGAGCTCTCAAA
CATGTCTCTAAGTACAACTGGATCACCAGGGTGCACCCATTAACCTGCCCCCCTCCTCCAACAGTCTGCCCCCT
GGTTGATATAAGACATACATCAGTAAGAAATACCAGAGTATAATAAGAGGTAAAATGATTGATTTGAAGCACTG
GTGAGCACTCCTTTAAAGGGAGTGCCAGTGATAATGTTGCCAGTGTTAATAAGCCGTAACACAAGCACAGGGAA
TTTCATTTCTTAGTAATATTTTGGCAAAGCCTCTCAATCCATTACTTTTGACTCAAAGGGCAGGATTTTCAAAA
GTTAATAAATGATAACTCCAGTGTTAACAAATCAGTATGTAGCGTTGATTTTATGCCTATCTCGTTATTAAATA
ATCATGCTAACGTGATTTCCAAAATTATGTTGATTTATGAAATAATGTTACTGTCTTAACGAGCAGTGCTTCTT
GCAACTATTTCTTTTGTTTGCTTGGAAATTTAAAAGGAAGTCCGTGTTAATTTTCATAATTTATCAGGAGTTAA
TTTGCACTTGGAAAAGGAGGGTTTTAATTAATGAAAGACTATAACTCACCTTGAAACAGAAATTTAACAGACCG
CGGCTGTGATGCCGATGATACAGAGACTACCTAGATAATTCAAAATTTGTAGTTTTGAAAATATTTTAGACTTT
TATATAGTTGAACATAAGAAGAACATAAGAAAGTTTACAAACGAGAGGAGTGTTACTGATTTAGTCTCACAGCT
GTGTGTGTTTGTATGTTACTGATATATTAGTTTTTCTTGGAATGATTTCTAAAGTTCAAATTCCTGGAATAGTT
TTTTTTCCAGCTTGACCCTCAAAGCCACCTCTCTCACGCTGAATTATATATCTACATCTGTTTTTCTAAATAAC
ACACACACGCAAAAAAACTTAAGAACATGAGAACATAAGAAAGTTCGGCCCATCTTGCTCGTTTGGTTGTTAGT
AGCTTATTGATCCCAGAATCTCATCAAGCAGCTTCTTGAAGGATCCCAGGGTGTCAGCTTCAACAACATTGTGG
TTATGAATGAGATTTATTCACTTGTTTTAAAAATTTCTAACCTTAAACCCGGAACACTGCCCGATGAAATCGCA
GGAAGGGATGCATGGATGCATAAAATGCAGTGGCAATGGGGTGACTCAATAGGTGAAAAGATAAGGGTATGGCA
GTACTAATAAATATCTACAATTTTGGCCAGAATTTAAGGCGACGTGATCGTGAAAACCACATATGACTGGTGAC
ATGCACGTATTCTCTCTATTATTATTATGAGTTTATTTAGCAGACGCCTTTATCCAAGGCGACTTACAGAGACT
AGGGTGTGTGAACTATGCATCAGCTGCAGAGTCACTTACAATTACGTCTCACCCGAAAGACGGAGCACAAGGAG
GTTAAGTGACTTGCTCAGGGTCACACAATGAGTCAGTGGATTTGAACCGGGGACCTCCTGGTTACAAGGCCGTT
TCTTTAACCACTGGACCACACAGCCTCCCTATTAAATACACACACACACACATACATACACATACATACATACA
TACACACATGGTATTCTATTGGTTTGTATATAATGTCCTTGCCAATATTTTCTGAAAACAAGGCCTGACAACCC
CACATTGTACTTTTTAACTATTCGACAAATTATTTACAGAATAAAAACTGCGTGATTCATTTATTATTAACAGC
GTTATCATTAAATTATACCAAATTTTTATTTACAATGAAGGGAACCTATACTGTGTCGGCTAAATGCTTATAAA
GTCAGCAAAAACATGTATTACATTTTCTGTTGATTGTGAATAAAAACCGTGGTTTGGGTATAATTTAATTATAA
TGCTATTAATAATAAACAAATCATGCAGCATGGTCCTAATAGCAGTTGTTCTGTAAACATTTTGTCTAATAGTT
AAAAGTGCATATGGGGCTGTCAGGGCTTGTTTTCAAAAGATATCGCCACAGACATGATTGTATACAAACCAAAA
ACAATGTTGTTGTAATGTTGTCTCTCTCCCCACACTTTCTGTACAGTGTACACTCTCACCTTCACACGTCTGTA
TGCATCTATTATAAGATCTTCTTATAATACTGTAAGTGTTCAGTAGATGACAACATAAGAAAATAAATTCCAAC
CAATATACATAGATGGCTTACCCGGCCTATCCAACATATAAATAAAAAAAGTTCATTTATGTTTTTAACTGTGT
CGTCCAATTTTTACCCACACGCCAGATAATCGTAAATCTGCATTTTATATGTGAAGTTATCTAAATAAATATAC
ATTCTGTATGACTAGGGCAGCAGTGTGGAGTAGTGGTTAGGGCTCTGGACTCTTGACCGGAGGGTCGTGGGTTC
AATCCCAGGTGGGGGACACTGCTGCTGTACCCTTGAGCAAGGTACTTTACCTAGATTGCTCCAGTAAAAACACA
ACTGTATAAATGGGTAATTGTATGTAAAAATAATGTGATATCTTGTAACAATTGTAAATCGCCCTGGATAACGG
CGTCAGCTAAGAAATAAATAATAATGACAAAATGCACAATTTTAAATGAAGTTTGCAAATTAGAAGTTTACAGA
AAATAGATATTCCCATTATTTTGTATTTCCATTTGGCTGCTGCTCGAGCCGTCTCTCTGTACGGTGGTAGTTTC
CATAACAGCAAATTATTATTTTATTATTTGTTTATTTAGCAGACGCCTTTATCCAAGGCGACTTACAGAGTCTA
GGGTGCGTGAACTATGCATCAGCTGCAGAGTCACTTACAATTACGTCTCACCCGAAAAACAGAGCACAAGGAGG
TTAAGTGACTTGCTCAGGGTCACACAATGAGTCAGTGGCTGAGGTGGGATTTGAACTGGGGATCTCCTGGTTAC
AAGCCCTTTTCTTTAACCACTAGACCACACAGCCTCCTAATTATGCTTCCATTCATTTTTCTTCATCTCGTTAG

CATGCATTTTGATGAACAAGTTCCCCTTTTTTAGTCGTTGAGACAGGAAGTGATGTACAATTAAGCTTTTAATG
AGAAATGTGTTCATTAACAACCTCATTGACTCCATTTCAAAGTATTAACACATTTTGTTTGAAAATCTCTTGCT
ACTAAAGCTCTCGTTACTATATCACGAGTTTGACTGTTTTTTAAAATCCTGCCCTAAATCTCTTAAATTGCCAC
ACCTAGCCAGATGTACTGTACTGCAGCAGCTGCTGCACAGATGAAGCTTCCAGAAAATACAAAAAATGAGCAAG
AGGGTGGAGGAGGTTGAACTGAGTTCAGCAATCCCCATTGTTTAACAAAGATAACAAATACAAAAAATATATGT
TGGCAGTGATCTGATTAGAACTGTGTTATTCTGTTGCTCTCTACAAAAACAAGAGTACGAACAGAGCCGACGCA
TTGAAAGGCCTCCACACTGCACTCTAATGGGAGGCTGCAGGCTGCACATACTGCACACAGATTTAATTTGTGTT
TTCTACCAGTCTCAGTTTGCAGTTGGGTGTCACTAATTCTGTATTTGTTTAGGAACTGCCGGTGTTTTGGGTGA
ATTTCCTGTTCAGGGAACTGAAACAATCTGATTTGAACTGAGTTTTAATTTGTTCTGCCTCAGTATGAATTGTA
CTATTTCACCAAGGCAGATCCCAGTGTTTGGAACAGTGTATTTTCACAAGGAGGGCTGTATAAATCACACTCTT
GAATTTGAACATTACCTTTTGCATTACTGCTGGACAGAATGGAGTTAGCTCTCTTTGAGATTGCTCTTTATAAT
ATTGTTTCTGTATCTTGTTTTTCAGATGACACAGTGTTTATGGATGATGAGAAATTCTTAAAGGAATATGTCCT
GAACGAAACTGGGAAAATTTACTACGGGACCGAAAAACAGATTGGAGCTCGAACCTGGAACTTTGGACAGGTCT
GTGTGTCTGTCTGTCTGTCTACATGGCTGCATGTGGGTGATCCTGCTGACTATGCAAATTACACATTTAAATGC
TGTGTTTCAGTACAGTACAAGTACCTGCCTGTCTGTCTGTCTGTCTGGTCTGTCTATCTGTCTCTATAATGTCC
AGGTTATGGATTGAAGGGAAATGTCAAATTAGCATTTTATTTATATATATATATACTCTAAACACACTCTTAAA
CTCACACAGACTCATTCGATTTGAAGTTTTAATGAATCAGAGTAGTATTAGTACCCCTTCGGTTTATTAAATGC
TAAAAAATGGATTGAGTAGTTATAGACTATGTCGAATCCCAGCTGACAGGCAATCTGGGCAGTCCAGTGCCTTA
ACAGGTGTTAGCATTAATAGTTCAATACATTTATGGTTACAACTTGTTTTACCCTAAGGCTACGCATGCATAAA
ACTAAAAACGCCCACCGCTAAGTCCTAAAACATCCAATATATCAATATCATAGACTAAAACATAAGTCATATAC
CTTATAGCAATGCATCAATATAAGAGAGATATAGAATCCAAATAGTTCTTACCTCCCAAATTATATATGGAAGT
GTAGAGTATAAGGTAAGAACAGGAACTGTCTTCAAAGGCAAAGACTTCTGAACACGACCCCAACAGCAATCAGC
TCGATCAGAGATCTTCAGAGCATGGATCACACAACTTGTAGTTAGCAAGTTGGTGACACTTGACTAAGATTTTA
CCTTGGTTTTTATAGAGTTTTTGCTTCGCTTTTTACGTCAGAAGTCTCAATGAAGTCTTAAACATTAATCAGCC
TTAACAGCTCCTGTCTCAAGTCAGCAACATTCTCAAAACACCCGGTAACTCCCTCCATAAAATGAATTAGACGT
CATCCCCTAGTCCACCTCTTCTACATCTCCGAAATATGTAAGGTGAACTTTTCAACTGATGTTATTCTTTCTTG
GTACCAGAAGGTATTTCTTATATGTAAAAGATCACTCACTATAGCAACCTTGGATTCCAAACCCACCAAACATA
ACCCTCCGTCTGGTACTGTAAACAAGCAAGGGTTACCAAGATTAATTACCTTCTCTTTGGCAAGCCTACTGTAT
GCCTGACACAGACTGATACCATTCTGCTTTGACTGAAGTTTTTACTGTAAGTTCATTTCTGCACTATATTCTCT
TAGTTTCAAACATTTCCAGTTCTTCTGTTAAAATAAAACATTTACACTTATATTCTGTTTGAATCATTCTAGAA
TACCTGTGTTTAGCTTAAAGACTACTTTCAGATCAAGCTTACTTCTTTAAATAAGAGACTTGATGCTCACACCT
GCCTTTCTGCTCAAGGTTATAATAGAATAAACAGGAATATAGAATGCTCTAACATAACTATAGGTTAATACAGA
GCATATTGTAACTTTTTTGTATGAACAGTGTGTTTTAACAATTTGTTTAGTATTTCTGCTGGCTTTATATTCTT
TAAAACACAGTACAGTTATTACAACATTTTAACACACATGCAGTTTCACAAATGTTCAATTTGCATCCCAAAGA
CCAGCCTGCTTCCAGTATCTGTCACAGTCTTTGATCAGTTTCTGCTCTCAGTGAGTCTGTCAAAGCCACCACAG
GTGTGTCTTTAAAATCCTGATACCTGCAAAAACTTAAATCTTGTTAGCTGGGCTCCCATCTCTACATTCCCATT
CCTATCATCAGTGAGCAAAAACATTTTAACTGAATCTGGGTCCATCAAAGAACCGCACACCCTTGCAACCGAGG
CCTTCCATGCAAAAGGATCTAATGACTTATAGCACAAAAGGGACGTGCCAGTGCCGTCTGTGACAGAATGCTTA
ATTACAATCACCAACTGTCTGGTGTCCTGGAGGCCTGTCTGTCTCTCTGTCCATGTGTGTCTGTCTGTCTGTCC
ATGTATGTGTCTGGGTGTCTCTCTCTGTTTATCTCTCTCTGTGTTGCAGTGTGTATGTGTGTGTATATATATAT
ATATATATATATATATATATATATATATATATATATATNNNNNNNNNNTATATATATATATATATATATATATATA
TATATATATATATATATATCCTCTTTCTCTGTGTCTCTCAGTTTGATGCCGGTGTTCTAGAGGCCTGCCTGTTT
ATTCTGGATCGCAGTGGGGTGCCTCACACTGGAAGGGGGGACCCAGTCAATGTGGTCCGAGTCATCTCTGCCTT
GGTAACAATTATTGCATTTTTATACTTTTTATTCTGAGCTGCAGTTCCTCATTGTGCCTGTACAGAGTGAGGGG
GCGCTCTCTCTTGCTTACGATATTATTATTAGTACAGTCGTCTCCGGCTAAGAGGACACCCCTCAGGAAGCAAG
CGAAGTGTTCTTATAGCCGAAGTGTTCTCTAAGACGGAGTTAGCCAACAGACACAATATGAATGAATCAAGAGT
GAAGACATGCACAAAAATTATTTAACAGAATGGTGAAACAACTCACCAGAACAGGAAACACCAAATAGCTTGGC
GACTTGTGCAATTTCCAACCTTTTATAGCAAGAGAAACAGTGATGCATTTCACCGTTTTGTTTACATGCCATTT
TGTAGCGGTGAGGTGCACTCGTGGAAATCAGAATGCAAAACAAGGCAGTGGTATGACGGCAGTTCTGGAAAGAT
TTAATCAATCAGTGAGCCTCAAGATACTCTCGTGATGACAAGTCACATTTGAAAAGACCAAACCATTTGAATTT
AAGTTTAATTATGATGAGTTATCAGGTTACACAACAGCACTGCATCCGTTGTGAACGAATCACTATCGGTGCCA
AAAAGGTGTTCTTTTAAGCAAAGTTCCTGTTCCTTTAGGTGGAGCATTTATAATGGAAAAAATCTGTTTCAGCA
CAAGGGTGTTCTCTTAGCCAAAGTGTTCTCTTAGGAGGTGTTCCTATACGCAGAGACTACTGTAGTAGTGGTAC
TAGGAGTTTTATTCTTATCATTATTATTATTACACATTTCTATTATTCTGAGCTGCAGTTCTCCGTTGTGCCTG

TACGGAGTGAGGGGGCGCTGTATTTTATTTGTAGTATTTTCAGTAGTAGCAGTAGTGGCAGCAGCAGTGTTATT
ATTGATCTTACTATGATTCTGAGCTGCAGTTCCTGTAGGCTGTCTCCTGCTCACTGTGTTATTATTCTGATTCC
CCCCTCTCCCAGGTGAACTCGATGGATGAGGATGGAGTCCTGGTTGGGAACTGGTCAGGTGATTACTCGCTGGG
CACGGCTCCCACAGCCTGGTCCGGCAGTGTGGATATCCTGACCCAGTACCATCGGAAGGGGGGCAAGCCGGTCT
CATACGGGCAGTGCTGGGTCTTTTCAGGGGTCACCACCACAGGTTTATTTTTATTTATTTATTTGCATTTTTAT
ACAGTAGTATCTTAAAGCACTGTACAGTACATAGTAGAAAATCAAACCACAATACATTTGTAAAACATCTACCA
TAATCATACCTGTTTAATAACATAATAACATTCAAACGGTATAATAAAATAATAAATATATATACACACATGCA
TACACAGATAAATATACTTACATACACACATCATCAACCTCATAATAATAATAATAGAAATCCAGCAATATGGG
GCTCCCGAGTGGCGCATCCAGTAAAAGCACTCGCTAGAGTGCAGGATGCGCTCTATAGCCTGGACGTCGCGAGT
TCGAATCCAGGCTATTCCACAGCCGACCATGGACGGGAGCTCCCAGGGGGCGGCGCTCAATTGGCCGAGCGTCG
CCCGGGGGGAGGGAGGGTTTGATCGGCCAGGGTGTTCTCGGCTCACTGCGCACCAGCGACCCCTGTAGTCTGGC
TGGGCGCCTGCGGGCTTGCCTGTAAGCTGCCCAGAGCTGCGTTGTCCTCCGACGCTGTAGCTCTGAGGCGGCTG
CACGGTGAGTCTGCAGAGTGTAAAGAAGCGGGCGGCTGACGGCACACGCTTCGGAGGACAGCGTGTGTTGATCT
TCGCCCCTCCCAAGTCAGCGCAGGGGTGGTAGCGGTGAGCTGAGCCTAAAAATAATTGGGCATTTCAAATTGGG
GAGAAAATAATAAAAAAATAATTGGCAACGACTAAATTTAAAAAAAAAAAAAAAAAAAAAGAAATGCAGCAATAT
AAAAGTTACATTAAAACCCACTAAGACAATAAAGCTGTTTTATAAAAGTACATTTTCAGTCTTGACTTAAAAAC
TGTAATGGTCCCAACTTCCCTGATAGACACAGGCAGAGCATTCCACAATTTTACAATTTACAAGAAAAAACCCT
GATTTTGTTGACCCTAGGTCAGTCTGTCTGCCTGTCTCTGTGTGTGTGTGTCTCTCTGTGTGTTTGTGTGTCTC
TGTGAGTGTGTGTGTGTGTGTGTCTCTGTCTCTGTGTTTGTGTGTCTCTGTCTGTGTGTGTGTGTGTGTTTGTG
TGTCTCTGTCTCTGTGTGTGTGTGTCTCTGTCTCTGTGTGTGTGTGTCTCTATGTGTGTGTGTTTGTGTGTCTC
TGTCTGTGTGTGTGTGTCTCTCTGTGTGTTTGTTTGTGTGTCTCTGTGTGTTTGTGNNNNNNNNNNTCTGTGTC
TCTGTCTCTGTGTCTGTGTGTCTCTGTCTGTGTGTTTGTATGTCTCTGTCTGTGTGTGTCTCTGTCTCTGTGTG
TGTGTGTGTGTGTGTGTCTCTCTGTGTGTTTGTGTGTCTCTGTCTGTGTGTGTGTGTGTGTCTCTGTCTGTGTG
TGTGTGTGTCTCTGTCTGTGTGTGGAGTGACTGGAGTCAGACGCACAGTTCTCACAGTGCGCTCCTCTCTCCCC
CTCTCTCTCCCCGCAGTGCTGCGTTGTTTAGGGATCCCCACGCGCAGCGTCACCAATTTCTGCTCGGCTCACGA
CACTGACGTGTCTCTCACCACGGACATCTACTTCGATGAGAACATGGAGCCCCTGGCGCACCTCAACATGGACT
CCATCTGGTCAGTACCGCAGGCCTTGCTGCTGGTCCAGTCTGTTTACTGCCTTTCTTCTTGCTGCTGTTACTCA
AACATTAACAGAACAAGGACAAGATCAGCTACACAAGCTGCCCTATATCACAGGCCCCTAATACCCTATCCCAG
TACCATATACCCTATCTCCTATACCCTGCGCCCTACCCCTATCCCAAATTCCCTACACAATATACCATACTCCT
TTTACCATATCCTCTATACCCTGCCCCCAATAGCCTATACCCTACCCCATATATCTTACCCCCTATCCCCAATT
CCCTATCCCCTACCCCCTACCCCCATGCCCTATGCTGGTCGTGGTGTCGATGCTAGTGTTCTGTCTGCTTTGAT
AGTTTCAGGATCTTGTGTACAATGTGTGGAGTGAGTGCTGGGTGAGTGGAGGTCCACGTGTCTGTGTGTGGCAG
TGCTGTGGGGTGTTCATGGTTGTTTGTGGAGTGGAGTGAATGTGGAGTTAGTGCTGGATGGTAGTGCTGCTGTG
TTGATATGTTATCTGTTGTATTGTATCTTAATTTACCACAGGAACTTCCACGTGTGGAATGACTGTTGGATGGC
TCGACCGGACCTGCCCCCGGGGATGGGGGGCTGGCAGGCGGTGGACGCGACGCCCCAGGAGACGAGCCAGGGGA
CCTTCTGCTGCGGCCCCTCCTCCGTCATAGCAATCCGCAACGGACTGGTGTACTACACCCACGACACACCCTTC
ATATTCGCTGAGGTGTGGACACAATACACAGTACTACTGCATCCTACACCCTGCACTGCACCCCTGCACCGTGC
ACACTGCACCCCTGCACTCCTGCACACTGCAACCTGTACACTGCACCCCTGCACCCTGCACCCTGCANNNNNNN
NNNCTGCAACCTGTACACTGCACCCCTGCACCCTGCACCCTACACCCTGCACCCCTTCACCCCTGCACCCTGGT
GTACTACACTCACGACACATACCCTATAGCCTCTTCCCTACCCTCTCTTCTGTACAGATGTCCTATATTCTCTG
ACCGATCAGTGGACTAACACTGACTGGTTCACACCAAGTTGCAATCGAGGGTTATAGGATCTGTGTGTACGGGT
AATATCACAGCCACTCACCGTCCTAGAACCCTTTTGTATTCAGCTTTATCGCTGACTGGCTGGAAGGTCCTCAG
CACTGTAATTGACTCTCTCCTCATTCTCACGGCAGGTGAACAGTGATAAGATCTACTGGCAGCGGCAGGCGGAT
GGCTCCTTCACCAAGGTGTTCAGTGAGAACAAGGCAGTGGGGCGCTGCATCAGCACCAAGGCAGTGGGGTCTGA
GGAGAGGGAGGACATCACTGACTTGTACAAGCACCCTGAGGGTGAGAGAACGGAGAGAAGGTCTAGCAGTACAG
GGCTGAGTCAGCCCAGGACTATAGAACAGCAAAGTGCATTAGAGTACTGCACGGTATATTGTGATATACTATAG
TACAGTATATTGTAATATAGTACAATACAGCATGGTATAGCATAAGATATTATACAGGATGATATTAAATCTCT
CTGTCTCTCGGTTGCTAGGCAGCGAGGAGGAGCGCATTGCCGTGGAGACGGCCTGTCGCTACGGCAGCAAGCCC
GATGTGTACTCGATCCGCCAGGCGGAGGACGTCAGCGTGGAAGTCAGCACGGATGGGGGGGAGCTACAGATGGG
CCACAACGCCTCAATCCGCATCACCACGAGCAACAAGAGCCAATCCGCACGCAGCGCCGTGCTGCACGGCCAGA
TCTCAGTCATGTACTACACCGGGGTCATCAAGGCAACCGTCAAGAAGGACATCATCAACATTGACTTGCTGCCC
GGCGAGGGTAAGAAACTGGGGCCATACTTACTGGGATTTGTACACGGAACGGACCCTTGCAGCAGAGCCAAAAT
GGTGTCAGTTTCACTGTCATTAAAATACTCCTCTAGAACAGAGGTAACATTAAGAACATAAGAACATAAGAAAG
TTTACAAACGAGAGGAGGCCATTCAGCCCATCTTGCATCTTGGAAGTTAAGTGGAGATAGACTAGAGGGAAGGA

GACACTTCTTCACACAGAGAGTGGTGAGGGGATGGAATGGGCTACCTAGGGTTATCTGGTCGTGTTGTTGATGC
TGAATCACTGAGATCAATCAGCTACTAGGAGCCAGACAAGCTCTGATGGGACGAGCTCTAATGGGACGAGCTCT
GATGGGACGAGCTCTGATGGAACGAGCTCTGATGGCCTCCTCTCATTCAGGAATTTTCTTACATTCTTATACTT
AACGAACAGCATAAGGTAGCACTCCAAAAACACCCTATCCTTCCCACTTTCCTGACTCTGAAATATCTTGCATC
AAATATAGAATGGGCTTTTATCCAAAAAAAAAAACAATTAAAAAAACTTACGTTAAAAAGGTCTCCCGTAGTAA
AAAAAAAGGAAAGTGTAATAAAGCACTGTGAAAGCATGATAAAGCATAGGGAAGCATTGTAAAGCACAGAGAGG
TNNNNNNNNNGAGAGGTGTGGTAAAGCATAGGGAAGCATTGTAAAGCACAGAGAGGTCTGGTAAAGCATAGGG
AAGCATTGTAAAGCACAGAGAGGTATGGTGTGTGTTATTGACTGCGTGTGTCCTGTGCTTGCTCACACAGAGAA
GACAGTGGAGTGGGTTCTGACGTACGCGGACTATCAGGACCAGCTGGTTGACCAGGCTGCTCTCGTGATGACTG
TGGCGGGTCGGGTCAGCCAGTCCGGTCAGGTTCTGGCCACTCAGCATGTCTTCAGACTGCGGACTCCGGACCTG
CAGATCCAGGTACCAGCCGGGCTCAGGAATCCCAGAGCGATACTGCACTGAATTTATATAGATATATTGTGAAC
GTAGCCTACTGGCTGAGGCTCACTTTTCACCCTTACACACAGAGCCAGACACAGAATGGTATTTTAATAGCACT
TTTGTGCACTTTTATTAATTACAATAACAAAATAAACAAACAAAAACTTCGGGGTGCTAACTAACCTTTCTTCC
AGTTCTTAACAATGGCTGGATGGCTAAGCCGTTTACCAGTCAAAACTAAACACTTATCACACACACAGCAGGTT
CACACAGTAACTCCTTCGACCACACAGGTCTCTCCATCAGCAGCCCAGACTGTAGGGCTTCCCCTGCCCTTATG
CAGGGCTTAATCAGCCTCAGGTGCTTCTCATTTCCTTTATTCCTGTGGCATTCTGGGAAATGTAGTCCCTGTAA
TCCCTCCTGGACTACACTTTTTCTCCTTTCCCCTGTCTGCCACACATCCTTCGTCTTCTCTCTCTCTCTATAAA
TCTATATATTTATATATCTATATCTATATCTATACACACACTGTGTTAATGCACTGTGCTATTGCACTGAATTA
TATATAAATGTACAGTGTTTTAATGCATTGTGATATTGCACTGAATATCCTTTGGATGAGAAGTAAAACCGAGG
TCCTATTGTAGTGACTCTGCAGCGACAGTTGTTAATGCATAATTTACCCCCTAGTCTCTGCAAGTCACTTTGGA
TAAAAGCATCTGCTAAATGACTAATTCATAATATATAGTGTGTTACTGCACTGTGATATTGTGCTGTAATATAC
AAACCATGCCTATTACACATTCAATAGCATTGAGTTAAGAAATACGAAAGGAAACTTAGAGTTCAGTGTCAGAT
GTTTCCACTAGAAGTCTTTTCAATGTCTGCAATACGCTGCACTTTTCTCCTCCAGCCTGAAGGCGGTGCTGTGG
TGGGTCAGGAGATGAAAGCCAAAATCATCTTCACCAACCCACTGCCCAAAACACTGAAGAGCGCTGTGATCAGT
GTGGAGGGTCCGGGCCTGCAGACGCCCAAGAGAATCAACATCGGGTAAGAGAAGCGAAGAGCCGTCTGTCAGTC
TGTATGTGTGTGCGTGTTTATCAGTGTGCATGTGTGTGTGTGTCTATATATATATATGTGTGTGTGTGAGTG
TCTGTGTGAGTTCAAAACAATGTGCAGACTTCTCACAAAGGCGTTCAAACAGAACCAAAAACATGCCTGTGCTG
TAATTCAATCCACGCTACAAACTGGACACAAGGACTATTAATCCATCAGTGATTGTTTAATTGAGACTTGGCAA
AATAAAAGCCGACAATCAATAACAGATTAATCAAATGAATGGATTGATCTCTGCTTTCTGTGTGTGTGTGTGTG
TGTGCCTGTCTGTCTGATGAATCACTATCTAATAAGAGCCGGTACGCATTGTCTGTCTGTCTGTCTCTCAACTC
CTCTCCTCTCATCTTTTAATATTTTCTTTGTTTTCCCTCCCTCAGCGATGTGGCACGTCACTCTACAATCACCC
TGACAGAAACGTTTGTTCCTGCCAAATCTGGCCCTCGCAAGCTCATCGCCAACCTGGACTGCAGGCAGCTGACC
CAGGTTCACGGGGTCGCAGAGTTCATAGTTCAAGACGAG

**TGM1B sterlet (accession XP_034770304.1) interrogation.** The genomic sequence was obtained as a set of mostly short alignments.

**Exons 3-7 (partial)**

TCCTTGTGTTCTCTTTGCAGATCAGATACTGTCGGTAAAGGCCGTTGATTTGCTGAAATCGCGCAGCGGAATTA
ACCGGCAGTCGCATCACACCGACGAATTTGAATACGATGACCTCATCCTGCGCCGTGGCCAATCGTTCCTCCTG
GAAATCGAATTCTCGCGACCTTTCAACCCCGACACAGACACGGTGCACTTGGAGCTGCAGATTGGTGAGTGGCT
GTCTTTATAATAGTAAGGGATAGCTGACATATTGCTAGTGAATTGAATTTACACTGAAGACGCTGAGAAAGACT
TCTAGTGGAAACGTTTGCCATTGAATTTACACTGAAGACGCTGAGAAAGACTTCTAGTGGAAACGTTTGCCATT
GAATTTACACTGAAGGCACTGAGAAAAGCACTGAAGACAGCGAAGAATTAAATATATATATATATATTTATTTT
TATTTTCAGGTCCTCTGCCACAGGTGTCTAAAGGCACCCACGTCATCATACCATTGGTCAGGGAGCTGGAAGAC
AACCAATGGGAAGCCAAGATTGTGCAGAGGGCGGGGAGTAGGGTGAAAGTGTCTGTCAGGTCTCCGGCGACAGC
TGTGATTGGCCGGTACAAGTTTGCAGTGACCACTCACAGCTCGAGGGGAGACTTCAAGATGGAACACGACCCCA
AGAACGACATCACCTTCCTGTTCAACCCCTGGTGTGAAAGTGAGTGTTGTCCCCCCTGCAACGAGGCTCGGTTT
TGTTTGGGAAGATTGGTTCCTACTTGAAAGAAATCTGAGCAGATGGTATCTGAACACAGAAAGAAAGAAAGAAA
TAGAAAGAAAGAAAGAAAGAAAGAAAGAAAGAAAGAAAGAAAGAAAGAAAGAAAGAAAGAATTTGTTTTTTTTA
GCCCTTCCTTGTCTCACACTGAAATAAGAATCTGTTTCTGAATGTTTAGATAAGAGACTTTTATTCAAGTTTAA
AAAGCCGAGCCGCGTGGCCTCGTGGAGCTGTGTGGGTGACAGTGTTTGTGGCTCCTGGTTCTCAGGTGACACGG
TGTTCATAGAGGATGAAGAGCTCCTGAAGGAATATATCCTGAACGAAACGGGGAAAATTTACTACGGCACTGAA
AAGCAGATCACAGCTCGAACCTGGAACTTTGGACAGGTGTGTGTGTATCTGTGTGTGTGTGTCTGTGTGTGTGT

GTGTCTGTGTGTGTGTCTGTATTTCTGTGTGTGTGTCTGTCTGTGTGTCTGTCTACGTGTCTGTGTTTCTGTCT
GTGTGTCTGTCTGTCTGTCTGTGTGTGTGTCTGTGTGTGTGTGTCTGTCTGTCTGTGTGGCTGTGTGTGTGTGT
CTGTATTTCTGTGTGTCTGTCTGTGTGTCTGTGTGTGTGTATTTCTGTGTGTGTGTGTGTCTGTGTGTGTCTGT
ATTTCTCTGTGTGTGTGTGTCTGTCTGTCTGTCTGTGTCTGTCTGTCTTTCGGTGTGTGTGTGTATCCCTGTCT
CTCAGTATTGCAGTGTGTATATATAGATATATATTCTCTCTCTCTCTCTCTCTCTCTCTCTCTCTCTCTCTCTC
TCTCTCTNNNNNNNNNNCTCTCTCTCTCTCTCTCTCTCTCTCTCTCTCTCTCTCTCTCTCTCTCTCAGTTTGATGC
TGGTATTCTAGATGCCTGCCTGTTTGTTCTGGATCGCAGTCAGATGCCTCACTCCGGAAGGGGGAATCCAGTCA
ATCTGGTCCGGGTCATCTCTGCCTTGGTAACAATATCTCTTAAAATGTTCATCCTTTTTGTTTTTATTGTGAGC
TGCAGTTCCATGTTCTGCCTATTCGGAATGAGGGGGCGCTGTCTCTCCTGCTTACTGTATGTTTATAGCAGTAG
CTGTGGTACTTGCAGTTTTATTATTATTATTATTATTATTATTATTATTATTATTATTATTATTATTATTATTA
TTNNNNNNNNNNTTATTATTATTATTATTATTATTATTATTATTATTATTATTATTATTGTATATTATATCCTA
AGCTGCAGTTCTTTGTAGTTCCAGTAGGCTCTCTATTACTCATTGTAATATAATAGTTATTATTATTGTGATTA
TTACCCCTTCCTCTCTCTCTCTCTCTCTCTCTCTCTCTCTCTCTGGTTGCTGTATTATAGATATTATTATTAGT
GTTCTGAGCTGCAGTTCCACGATGTGCCTGTACAGAGTGAGGGGGCGCTGTCTCTCACTTGGATTTTGCAGTGG
AGTTATATTACTATAATGACTGTGGTTGTGACTCCTCCCTCTCCCCCAGGTGAACTCGGTGGATGACAGGGGGG
TTGTGGTTGGGTGCTGGTCAGGTGATTTCTCCCAGGGG (overlap with the next alignment shown in purple)

### Exons 7(partial)-9

AGGGGGGTTGTGGTTGGGTGCTGGTCAGGTGATTTCTCCCAGGGGACAGCTCCCACAGCCTGGTCTGGCAGTGT
GGATATTCTGACCCGGTACCATCGCAGTGGGGGAGTGCCAGTACCTTTCGGACAGTGCTGGGTGTTCTCAGGGG
TCACCACTACAGGTCAGTCCGTCTGTGTGCGTGTGCGTGTGCGTGTGCGTGTGCGTGTGCGTGTGCGCGTGCGC
GCGTGCGCGCGTGCGCGCGTGCGTGCGTGCGCGTGTGTGTGTGCTCGTGTGTGTGTGTGTGTGTGTGTGTGTGT
GCGTGTGTGTCTCAGTGTCAGTGTCTCTCTGTCTGTGTGTGTGTGTGTCTCTCTCTGTCTGTCTGTCTGTCTGT
CTTTCTGTGTGTCTCTCTGTCTCTGTCTGTCTGTGTGTGTGTGTCTGTCTGTCTGTCTGTCTGTCTGCCTGTGT
GTCTCTCTCTGTCTGTCTGTCTGTCTCTGTGTCTGTGTGTGTGTCTCTCTGTCTGTCTGTCTGTCTGTCTTTCT
GTGTGTCTCTCTGTCTCTGTCTGTCTGTCTGTGTGTGTCTATCTGTCTGTCTGTCTGTCTGTCTGTCTGTCTGT
CTGTGTGTGTTTCTGTCTGTCTGCCTGTGTGTGTCTGTCTGCCTGTGTGTCTGTCTGTGTTTGTGTTTCTGTAT
GTCTGTGTGTCTCTCTGTCTGTCTGTCTCTCTGTGTGAGTCTGTGTCTGTGTGTGTCTGTGTGTCTCTGTGCCT
CTNNNNNNNNNNCTCTCTCTCTCTCTCTCTCTCTCTCTCTCTCTCTCTCTCTCTCTCTCCCTCTCCCTCCCTCG
CTCCCCGCAGTGCTGCGTTGTTTAGGAATCCCCGCGCGCTGCGTCACCAACTTCTCCTCGGCGCACGACACCGA
CGTCTCTCTCACCATGGACCTGTACTTCGATGAGCACATGCGCCCCCTGACTCACCTGAACCAGGACTCTGTGT
GGTCGGTACCGCAGGCCCGGCTGATGGCCCGCTTCCTCCACTGCCTTTCTCCTTGAATGTGAACACCGAGCACC
CTATACCCTGTGCCCTACCCCCTACCCCCTATCCCCTATCCCCTATTCCTTACACAATATGCCCAATCCCCTAT
CCCGAACTCCCTACACAATATACCCTACCCTCATACCCTANNNNNNNNNNCCCCTATCCCGAACTCCCTACACA
ATATACCCTACCCTCATACCCTATCCCCTACCCCCTACCCCCTGTGCTGACTGTGAGGATTGAGGCTGGCGTTC
TGTCTGGTTTGATGTTGATTCACGATCTTGGGTACAATGTGCAGAGTTATTACAAAAATAACAATACATAATAA
TAATGACGATATCAATAAATGATAACAATATGAAATAATAAATAAAATTTAAAAAATAGTTGCAGAAAACAGGA
AATGGATAGGAATTACAAGAGTCAGGCCGGCAGGGCTGCAGGTTCGAGTTTATCGTGTCTGATTTTCGCCGCAG
GAACTACCACGTGTGGAATGATTGCTGGATGGCGCGTCCGGACCTGCCCCCGGGGATGGGGGGTGGCAGGCAG
TGGACGCCACGCCCCAGGAGACCAGCCAGGGGGTCACTTGCTGTGGCCCCGCCCCCGTCCAGGCGATCCGCGAC
GGACTGGTGTACCACAACCACGACACACGCTTCATATTCGCAGAGGTGACG

### Exon 10

CCTCTCTCTCCGGCAGGTGAACAGTGATAAGATCTACTGGCAGCGGCGGGCGGACGGCTCCTTCACTCAGGTGT
TCAGTGAGAGGAAGGCGATCGGGAGCCACATCAGCACCAAGGCAGTGGGGTCTGAGGAGAGGGAAGACATCACA
GACATGTACAAATACCCTGAGGGT

### Exon 11

GGCAGCAAGGAGGAGCGCATCGCCGTGGAGACGGCCTGTCGCTACGGCAGCAAGCCCAACGTTTACTTGAACCG
GCAGGCGGAGGACGTCAGCGTTGAGGTCAGCACGGACGGGGCGGGGCTTCACGTGGGAAGCTCCGCCTCCATCC
TCATCGTCGTGGCGAACAGGAGCCAATCGGCACGCAGCGCCGTGCTGCACGGCCAGATCGCAGTCATGTACTAC
ACCGGGGTCGTCAAGGCAACCATCAAGAGGGACTCCATCAACGTCCAACTGATGCCCGGCGAGG

**Exon 12**

AGTCAGGTCAGTGCAGTGGGTTCTCACGTACTCGGACTATCAGGACCAGCTGGTGGACCAGGCTGCTCTGGTGA
TGACGGTGGCGGGTCGGGTCAGTCCGACTGGGCAGGTTCTGGCCACTCAGCACGTCTTCAGACTGCAGACTCCG
GACCTGCAGATCCAGGTACCAGCCGGGCCCAGGATTACCGGATCGGTACTG

**Exons 13-14**

CCTCCAGCCTGAAGGGAGCGCTGTGGTGGGTAAGCAGTTGCGAGTCAAAATCATCTTCACCAACCCGCTGTCCA
AAACACTGAAGAGCGCGGTGATCAGCTTGGAGGGGGCAGGCCTGCAGACCCCCAAGAGAATCAACATTGGGTAA
GAAAAAAACTGTGTGTGTGTCTGTGTGTGTGTGTGTGTGTGTGTGTGTGCGTGTGTGTGTGTCTGTGTGTCT
GTGTCTGTGTCTCTGTGCGTGTGTGTCTGTGTGTGTGTCTGTCTGTCTGTCTATCTGTCTGGTCTGTGTGTCTG
TCTGGTGTGTGTGTCTGTCTGTCTCTCAGTATCTCTCTTTGTTTAAAGTGTTCTGTTTTTTTTTTTTCCCTGCT
CAGCGACGTGGCGCGTCACTCCACACTCACCCTGACGGAAACGATTGTTCCTTTCAAATCTGGCCACCGAAAGC
TTATCGCCAACCTTCACTGCAGGCAGCTGACCCAGGTTCACGGGGTCGCGGAGATCACAGTTCGAGACCAATTG

## S2 Table. Primer sequences used for cloning, PCR and real time PCR.

| TGM1 Gene | Primer used for: | Sequence |
|---|---|---|
| 5' cloning and sequencing | | |
| A | cDNA Synthesis | GAAGTAGATGTCCGTGGTGAG |
| A | PCR | GACTCGGACCACATTGACTG |
| B | cDNA Synthesis | CCTCGCCGGGCATCAGTTGG |
| B | PCR | CTTCCGGAGTGAGGCATCTG |
| Real Time PCR (TaqMan Assay) | | |
| A | Forward primer | CAGACGCCCAAGAGAATCAAC |
| A | Reverse primer | TCTGTCAGGGTGATTGTAGAGTGA |
| A | Reporter | ATCGGCGATGTGGCACG |
| B | Forward primer | CGCGGTGATCAGCTTGGA |
| B | Reverse primer | TCAGGGTGAGTGTGGAGTGA |
| B | Reporter | ACGTCGCCAATGTTG |

## S3 Table. Nucleotide and amino acid sequences of white sturgeon TGM1A and TGM1B.

**TGM1A mRNA.** The ATG translation start site is shown in bold (yellow highlight).

CTAGAACTTCAAAACTGAAAG**ATG**CCTGCAAACAGCGTGTCAGTGCGGCGGCGCTCCGAGCTCGGGCGCT
GGCCGACGGTGAGTCTGGGGGGGGTCGGCGAGGAGGACGAGGTGACCCCCGGCACTGCTGGGGACTCGCA
GGTCGGGACGAAGAGGCAGGAGAGTCGCTGCAGTGGCTGGATGAGGAGAGTTTGCCCCTGTGTGTGCAGG
AAGAGCGCTGACGACCTGACCGACAACAGCGGCCCCACGGCAACCATAGAGGATGACAAGCGCACTATTC
CACTGTCAGATCTGATATTATCGGTAAAGAGTGTTGATTTGATTAAATCTCGGTCTGGAATTAACCGGAA
GGAGCATCACACAGACGAATTTGAATACGATGACCTCATCCTGCGCCGCGGCCAATCGTTCGTCATGGAA
ATCGAATTCTCGCGACCTTTCAACCCCGACAATGACACCATGCACTTGGAACTGCAGATCGGTCCACTGC
CACAGGTGTCAAAGGGCACCCATGTCATCATTCCATTGGTCGAGGAGCTGGAAGACAACCGATGGGAAGC
CAAGATTGTGCAGAGGGTGGGGACTAAGATGAAACTGTCTGTCGGATCCCCGGTGACAGCTGTGATTGGC
CGGTACAAGTTTGCAGTGGCCACTCACAGCTCGGGGGAGACTTCAAGATGGAACACAACCCCAAGAACG

ACATCACCTTCCTGTTCAACCCCTGGTGCGAAAATGACACAGTGTTTATGGATGATGAGAAATTCTTAAA
GGAATATGTCCTGAACGAAACTGGGAAAATTTACTACGGGACCGAAAAACAGATTGGAGCTCGAACCTGG
AACTTTGGACAGTTTGATGCCGGTGTTCTAGAGGCCTGCCTGTTTATTCTGGATCGCAGTGGGGTGCCTC
ACACTGGAAGGGGGGACCCAGTCAATGTGGTCCGAGTCATCTCTGCCTTGGTGAACTCGATGGATGAGGA
TGGAGTCCTGGTTGGGAACTGGTCAGGTGATTACTCGCTGGGCACGGCTCCCACAGCCTGGTCCGGCAGT
GTGGATATCCTGACCCAGTACCATCGGAAGGGGGGCAAGCCGGTCTCATACGGGCAGTGCTGGGTCTTTT
CAGGGGTCACCACCACAGTGCTGCGTTGTTTAGGGATCCCCACGCGCAGCGTCACCAATTTCTGCTCGGC
TCACGACACTGACGTGTCTCTCACCACGGACATCTACTTCGATGAGAACATGGAGCCCCTGGCGCACCTC
AACATGGACTCCATCTGGAACTTCCACGTGTGGAATGACTGTTGGATGGCTCGACCGGACCTGCCCCCGG
GGATGGGGGGCTGGCAGGCGGTGGACGCGACGCCCCAGGAGACGAGCCAGGGGACCTTCTGCTGCGGCCC
CTCCTCCGTCATAGCAATCCGCAACGGACTGGTGTACTACACCCACGACACACCCTTCATATTCGCTGAG
GTGAACAGTGATAAGATCTACTGGCAGCGGCAGGCGGATGGCTCCTTCACCAAGGTGTTCAGTGAGAACA
AGGCAGTGGGGCGCTGCATCAGCACCAAGGCAGTGGGGTCTGAGGAGAGGGAGGACATCACTGACTTGTA
CAAGCACCCTGAGGGCAGCGAGGAGGAGCGCATTGCCGTGGAGACGGCCTGTCGCTACGGCAGCAAGCCC
GATGTGTACTCGATCCGCCAGGCGGAGGACGTCAGCGTGGAAGTCAGCACGGATGGGGGGGAGCTACAGA
TGGGCCACAACGCCTCAATCCGCATCACCACGAGCAACAAGAGCCAATCCGCACGCAGCGCCGTGCTGCA
CGGCCAGATCTCAGTCATGTACTACACCGGGGTCATCAAGGCAACCGTCAAGAAGGACATCATCAACATT
GACTTGCTGCCCGGCGAGGAGAAGACAGTGGAGTGGGTTCTGACGTACGCGGACTATCAGGACCAGCTGG
TTGACCAGGCTGCTCTCGTGATGACTGTGGCGGGTCGGGTCAGCCAGTCCGGTCAGGTTCTGGCCACTCA
GCATGTCTTCAGACTGCGGACTCCGGACCTGCAGATCCAGCCTGAAGGCGGTGCTGTGGTGGGTCAGGAG
ATGAAAGCCAAAATCATCTTCACCAACCCACTGCCCAAAACACTGAAGAGCGCTGTGATCAGTGTGGAGG
GTCCGGGCCTGCAGACGCCCAAGAGAATCAACATCGGCGATGTGGCACGTCACTCTACAATCACCCTGAC
AGAAACGTTTGTTCCTGCCAAATCTGGCCCTCGCAAGCTCATCGCCAACCTGGACTGCAGGCAGCTGACC
CAGGTTCACGGGGTCGCAGAGTTCATAGTTCAAGACGAG

**Translation product.** The cysteine cluster (green) and active site region (red) are in color.

MPANSVSVRRRSELGRWPTVSLGGVGEEDEVTPGTAGDSQVGTKRQESRCSGWMRRVCPCVCRKSADDLT
DNSGPTATIEDDKRTIPLSDLILSVKSVDLIKSRSGINRKEHHTDEFEYDDLILRRGQSFVMEIEFSRPF
NPDNDTMHLELQIGPLPQVSKGTHVIIPLVEELEDNRWEAKIVQRVGTKMKLSVGSPVTAVIGRYKFAVA
THSSGGDFKMEHNPKNDITFLFNPWCENDTVFMDDEKFLKEYVLNETGKIYYGTEKQIGARTWNFGQFDA
GVLEACLFILDRSGVPHTGRGDPVNVVRVISALVNSMDEDGVLVGNWSGDYSLGTAPTAWSGSVDILTQY
HRKGGKPVSYGQCWVFSGVTTTVLRCLGIPTRSVTNFCSAHDTDVSLTTDIYFDENMEPLAHLNMDSIWN
FHVWNDCWMARPDLPPGMGGWQAVDATPQETSQGTFCCGPSSVIAIRNGLVYYTHDTPFIFAEVNSDKIY
WQRQADGSFTKVFSENKAVGRCISTKAVGSEEREDITDLYKHPEGSEEERIAVETACRYGSKPDVYSIRQ
AEDVSVEVSTDGGELQMGHNASIRITTSNKSQSARSAVLHGQISVMYYTGVIKATVKKDIINIDLLPGEE
KTVEWVLTYADYQDQLVDQAALVMTVAGRVSQSGQVLATQHVFRLRTPDLQIQPEGGAVVGQEMKAKIIF
TNPLPKTLKSAVISVEGPGLQTPKRINIGDVARHSTITLTETFVPAKSGPRKLIANLDCRQLTQVHGVAE
FIVQDE

**TGM1B mRNA.** The ATG translation start site is shown in bold.

TATTATTTTATAAAAAATAGGATTAGGGTTCGAATTTCAAACTGAAAATTCACACG**ATG**GCCGGGACTCG
CATGTCGGAGCCGCTTCACTCCGAGCTCGGGCGCTGGCCGACGGAGAGGTTCCGCGCGGATGTTGAGACG
ACTCCCGCCGCCGCCAGGGGCTCGCAAGGCGGGCTGAAGACGCAGGAGAGACGCTCTGCGGGCTGGATGG
AAACGGTATTCCCCTGTGTGTGCACGAATAGCGCAGACTCGCATGACGTGACCGACTACAATGTCCCCCC
AGCAACCAGGACGCCTGAGGATCCGCCGTCAGATCAGATACTGTCGGTAAAGGCCGTTGATTTGCTGAAA
TCGCGCAGCGGAATTAACCGGCAGTCGCATCACACCGACGAATTTGAATACGATGACCTCATCCTGCGCC
GTGGCCAATCGTTCCTCCTGGAAATCGAATTCTCGCGACCTTTCAACCCCGACACAGACACGGTGCACTT

GGAGCTGCAGATTGGTCCTCTGCCACAGGTGTCTAAAGGCACCCACGTCATCATACCATTGGTCAGGGAG
CTGGAAGACAACCAATGGGAAGCCAAGATTGTGCAGAGGGCGGGGAGTAGGGTGAAAGTGTCTGTCAGGT
CTCCGGCGACAGCTGTGATTGGCCGGTACAAGTTTGCAGTGACCACTCACAGCTCGAGGGGAGACTTCAA
GATGGAACACGACCCCAAGAACGACATCACCTTCCTGTTCAACCCCTGGTGTGAAAGTGACACGGTGTTC
ATAGAGGATGAAGAGCTCCTGAAGGAATATATCCTGAACGAAACGGGGAAAATTTACTACGGCACTGAAA
AGCAGATCACAGCTCGAACCTGGAACTTTGGACAG*TTTGATGCTGGTATTCTAGATGCCTGCCTGT*TTGT
TCTGGATCGCAGTCAGATGCCTCACTCCGGAAGGGGAATCCAGTCAATCTGGTCCGGGTCATCTCTGCC
TTGGTGAACTCGGTGGATGACAGGGGGGTTGTGGTTGGGTGCTGGTCAGGTGATTTCTCCCAGGGGACAG
CTCCCACAGCCTGGTCTGGCAGTGTGGATATTCTGACCCGGTACCATCGCAGTGGGGGAGTGCCAGTACC
TTTCGGACAGTGCTGGGTGTTCTCAGGGGTCACCACTACAGTGCTGCGTTGTTTAGGAATCCCCGCGCGC
TGCGTCACCAACTTCTCCTCGGCGCACGACACCGACGTCTCTCTCACCATGGACCTGTACTTCGATGAGC
ACATGCGCCCCCTGACTCACCTGAACCAGGACTCTGTGTGGTCGAACTACCACGTGTGGAATGATTGCTG
GATGGCGCGTCCGGACCTGCCCCCGGGGATGGGGGGGTGGCAGGCAGTGGACGCCACGCCCCAGGAGACC
AGCCAGGGGGTCACTTGCTGTGGCCCCGCCCCCGTCCAGGCGATCCGCGACGGACTGGTGTACCACAACC
ACGACACACGCTTCATATTCGCAGAGGTGAACAGTGATAAGATCTACTGGCAGCGGCGGGCGGACGGCTC
CTTCACTCAGGTGTTCAGTGAGAGGAAGGCGATCGGGAGCCACATCAGCACCAAGGCAGTGGGGTCTGAG
GAGAGGGAAGACATCACAGACATGTACAAATACCCTGAGGGCAGCAAGGAGGAGCGCATCGCCGTGGAGA
CGGCCTGTCGCTACGGCAGCAAGCCCAACGTTTACTTGAACCGGCAGGCGGAGGACGTCAGCGTTGAGGT
CAGCACGGACGGGGCGGGGCTTCACGTGGGAAGCTCCGCCTCCATCCTCATCGTCGTGGCGAACAGGAGC
CAATCGGCACGCAGCGCCGTGCTGCACGGCCAGATCGCAGTCATGTACTACACCGGGGTCGTCAAGGCAA
CCATCAAGAGGGACTCCATCAACGTCCAACTGATGCCCGGCGAGGTCAGGTCAGTGCAGTGGGTTCTCAC
GTACTCGGACTATCAGGACCAGCTGGTGGACCAGGCTGCTCTGGTGATGACGGTGGCGGGTCGGGTCAGT
CCGACTGGGCAGGTTCTGGCCACTCAGCACGTCTTCAGACTGCAGACTCCGGACCTGCAGATCCAGCCTG
AAGGGAGCGCTGTGGTGGGTAAGCAGTTGCGAGTCAAAATCATCTTCACCAACCCGCTGTCCAAAACACT
GAAGAGCGCGGTGATCAGCTTGGAGGGGGCAGGCCTGCAGACCCCCAAGAGAATCAACATTGGCGACGTG
GCGCGTCACTCCACACTCACCCTGACGGAAACGATTGTTCCTTTCAAATCTGGCCACCGAAAGCTTATCG
CCAACCTTCACTGCAGGCAGCTGACCCAGGTTCACGGGGTCGCGGAGATCACAGTTCGAGACCAATTG

**Translation product.** The cysteine cluster (green) and active site region (red) are in color.

MAGTRMSEPLHSELGRWPTERFRADVETTPAAARGSQGGLKTQERRSAGWMETVFP**CVC**TNSADSHDVTD
YNVPPATRTPEDPPSDQILSVKAVDLLKSRSGINRQSHHTDEFEYDDLILRRGQSFLLEIEFSRPFNPDT
DTVHLELQIGPLPQVSKGTHVIIPLVRELEDNQWEAKIVQRAGSRVKVSVRSPATAVIGRYKFAVTTHSS
RGDFKMEHDPKNDITFLFNPWCESDTVFIEDEELLKEYILNETGKIYYGTEKQITARTWNFGQFDAGILD
ACLFVLDRSQMPHSGRGNPVNLVRVISALVNSVDDRGVVVGCWSGDFSQGTAPTAWSGSVDILTRYHRSG
GVPVPFGQCWVFSGVTTTVLRCLGIPARCVTNFSSAHDTDVSLTMDLYFDEHMRPLTHLNQDSVWSNYHV
WNDCWMARPDLPPGMGGWQAVDATPQETSQGVTCCGPAPVQAIRDGLVYHNHDTRFIFAEVNSDKIYWQR
RADGSFTQVFSERKAIGSHISTKAVGSEEREDITDMYKYPEGSKEERIAVETACRYGSKPNVYLNRQAED
VSVEVSTDGAGLHVGSSASILIVVANRSQSARSAVLHGQIAVMYYTGVVKATIKRDSINVQLMPGEVRSV
QWVLTYSDYQDQLVDQAALVMTVAGRVSPTGQVLATQHVFRLQTPDLQIQPEGSAVVGKQLRVKIIFTNP
LSKTLKSAVISLEGAGLQTPKRINIGDVARHSTLTLTETIVPFKSGHRKLIANLHCRQLTQVHGVAEITV
RDQL

**S1 Fig. Independence of envelope formatting ability and cell density.** Passage 12 white sturgeon epidermal cells were grown in the absence of a feeder layer and sampled at the densities indicated. Parallel wells of 6 well plates were either trypsinized and counted or treated with X537A overnight and counted after treatment with SDS and DTT.

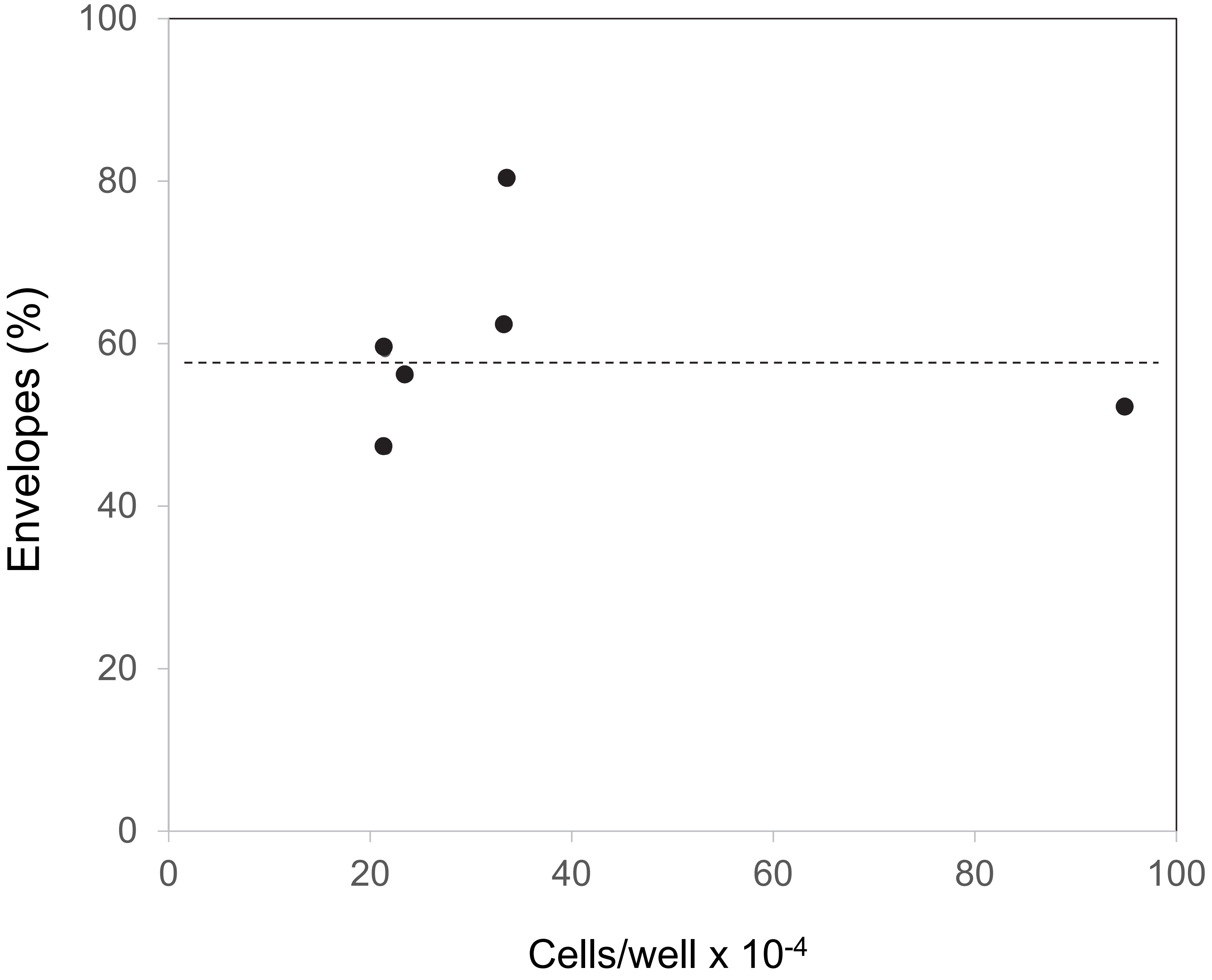

**Original Data**

**Table 1. Envelope formation.**

*Effects of cell suspension and ionophore treatment on envelope formation.* Parallel cultures were trypsinized, counted (Total), suspended overnight and counted again after addition of SDS and DTT (Suspended). Another set of parallel cultures was treated with ionophore overnight, then counted for envelopes after addition of SDS and DTT (X537A).

| | Sample 1 | Sample 2 | Sample 3 | Mean |
|---|---|---|---|---|
| Total | 945 | 718 | 765 | |
| % | 100 | 100 | 100 | |
| Suspended | 164 | 96 | 106 | |
| % | 17.4 | 13.4 | 13.9 | 15 ± 2 |
| X537A | 515 | 470 | 455 | |
| % | 54.5 | 65.5 | 59.5 | 60 ± 5 |

*Effect of cystamine, iodoacetamide (IA) and no treatment on envelope counts.* Each sample was a trio of parallel cultures treated overnight with X537A. One was treated with or without cystamine (20 mM) for 4 hr before X537A addition and another was treated with 20 mM iodoacetamide when X537A was added. After overnight incubation, the cultures were treated with SDS (2%) and DTE (25 mM) and envelopes in 10 µl in each culture were counted by hemacytometer.

| | Treatment | | | |
|---|---|---|---|---|
| Sample | X537A | X537A + Cystamine | X537A + IA | None |
| 1 | 346 | 3 | 5 | 1 |
| 2 | 296 | 2 | 3 | 2 |
| 3 | 348 | 4 | 4 | 0 |
| | Mean | ≤1% | ≤1% | <1% |

*Envelope formation by cells released in culture.* The floating cells were collected 4 days after the last medium change from two different confluent cultures, counted (Total) and counted again after treatment with SDS and DTE (Envelopes).

| Sample | Total | Wells | Cells/well | Envelopes | Wells | Envelopes/well | % |
|---|---|---|---|---|---|---|---|
| 1 | 330 | 6 | 55 | 89 | 8 | 11.1 | 15.4 |
| 2 | 633 | 8 | 79 | 98 | 8 | 12.2 | 20.2 |
| | | | | | | | Mean 18 ± 2 |

**Figure 3. Relative amounts of TGM1A and TGM1B as determined by real time PCR.** The ratios graphed were determined by the differences in Ct values and converted to antilog values.

| | Ct TGM1A | Ct TGM1B | Difference | Mean | SD | Antilog Mean | Antilog SD |
|---|---|---|---|---|---|---|---|
| P3 | 20.356 | 29.065 | 8.710 | 8.256 | 1.116 | 305.693 | 2.168 |
| | 21.364 | 27.817 | 6.454 | | | | |
| | 22.041 | 29.772 | 7.731 | | | | |
| | 18.966 | 29.031 | 10.066 | | | | |
| | 20.484 | 28.907 | 8.423 | | | | |
| | 19.066 | 26.802 | 7.736 | | | | |
| | 18.614 | 27.287 | 8.673 | | | | |
| P12 | 19.775 | 28.0978 | 8.323 | 8.026 | 0.288 | 260.629 | 1.221 |
| | 20.760 | 28.508 | 7.747 | | | | |
| | 21.079 | 29.086 | 8.008 | | | | |
| P15 | 19.863 | 26.780 | 6.917 | 6.927 | 0.438 | 121.686 | 1.355 |
| | 20.047 | 27.417 | 7.370 | | | | |
| | 20.206 | 26.701 | 6.494 | | | | |
| N8 | Mean of P3, P12, P15 | | | 7.736 | 0.441 | 213.231 | 1.358 |
| - - - | - - - | - - - | - - - | - - - | - - - | - - - | - - - |
| 8N | 19.943 | 24.380 | 4.437 | 4.518 | 0.167 | 22.910 | 1.123 |
| | 20.429 | 25.139 | 4.710 | | | | |
| | 20.352 | 24.759 | 4.407 | | | | |
| 8N | 21.590 | 25.425 | 3.835 | 4.159 | 0.334 | 17.867 | 1.260 |
| | 18.392 | 22.894 | 4.502 | | | | |
| | 18.133 | 22.274 | 4.141 | | | | |
| 12N | 18.153 | 24.948 | 6.795 | 6.098 | 0.987 | 68.484 | 1.981 |
| | 18.934 | 24.334 | 5.400 | | | | |
| GS | 19.555 | 23.909 | 4.355 | 4.623 | 0.379 | 24.635 | 1.301 |
| | 16.403 | 21.294 | 4.891 | | | | |

**Figure 4. Relative CYP1A1 Activities.** The samples were parallel cell cultures treated with the indicated concentrations of 7-ethoxyresorufin. In each case, the resorufin fluorescence values were normalized (Norm) to the TCDD response due to the variable response to TDDD among the cultures. Statistical calculations were performed using the normalized values.

| Human | Sample 1 | Sample 2 | Sample 3 | Norm 1 | Norm 2 | Norm 3 |
|---|---|---|---|---|---|---|
| Background | 51404 | 51404 | 58455 | 0 | 0 | 0 |
| Untreated | 140101 | 110869 | 209059 | 1.93 | 2.97 | 3.81 |
| TCDD | 4646648 | 2055428 | 4006156 | 100 | 100 | 100 |
| TCDD + 0.01 µM GNF351 | 3459244 | 1292466 | 2062090 | 74.16 | 61.93 | 50.75 |
| TCDD + 0.1 µM GNF351 | 139761 | 121451 | 71557 | 1.92 | 3.5 | 0.33 |
| TCDD + 1 µM GNF351 | 74679 | 70343 | 56971 | 0.51 | 0.95 | 0.04 |

| Sturgeon | Sample 1 | Sample 2 | Sample 3 | Norm 1 | Norm 2 | Norm 3 |
|---|---|---|---|---|---|---|
| Background | 46205 | 43031 | 64567 | 0 | 0 | 0 |
| Untreated | 68631 | 58029 | 96940 | 3.17 | 2.85 | 4.57 |
| TCDD | 753436 | 570020 | 773476 | 100 | 100 | 100 |
| TCDD + 0.1 µM CH223191 | 657462 | 570161 | 865137 | 86.43 | 100.03 | 112.93 |
| TCDD + 1 µM CH223191 | 513983 | 389468 | 644250 | 66.14 | 65.74 | 81.77 |
| TCDD + 10 µM CH223191 | 104670 | 49270 | 92792 | 8.27 | 1.18 | 3.98 |

| Sturgeon | Sample 1 | Sample 2 | Sample 3 | Norm 1 | Norm 2 | Norm 3 |
|---|---|---|---|---|---|---|
| Background | 64567 | 65543 | 55252 | 0 | 0 | 0 |
| Untreated | 96940 | 66932 | 73671 | 4.57 | 0.24 | 2.26 |
| TCDD | 773476 | 655778 | 758290 | 100 | 100 | 100 |
| TCDD + 0.01 µM GNF351 | 882572 | 687821 | 854193 | 115.39 | 105.43 | 113.64 |
| TCDD + 0.1 µM GNF351 | 790497 | 724806 | 817343 | 102.4 | 111.7 | 108.4 |
| TCDD + 1 µM GNF351 | 218431 | 309658 | 625158 | 21.7 | 41.36 | 81.06 |
| TCDD + 10 µM GNF351 | 70174 | 57474 | 69071 | 0.8 | 0 | 1.97 |

**Figure S1. Dependence of envelope formation on cell density.** On different days, pairs of parallel wells in a 6 well plate were trypsinized and counted (A) or treated overnight with X537A (B) and then counted after treatment with SDS and DTE. The cells and envelopes were counted using a hemacytometer, where the numbers of cells are tabulated per chamber unit (0.1 µl).

| Well | Cells | Units | Cells/unit | Vol | Cells/well x $10^{-4}$ |
|---|---|---|---|---|---|
| 1A | 864 | 8 | 108 | 0.2 | 22 |
| 2A | 1000 | 6 | 167 | 0.2 | 33 |
| 3A | 993 | 6 | 166 | 0.13 | 21.5 |
| 4A | 905 | 8 | 113 | 0.21 | 24 |
| 5A | 1174 | 8 | 147 | 0.23 | 34 |
| 6A | 2163 | 5 | 433 | 0.22 | 95 |

| Well | Envelopes | Units | Env/unit | Vol (ml) | Env/well x $10^{-4}$ | % Env |
|---|---|---|---|---|---|---|
| 1B | 404 | 4 | 101 | 0.1 | 10 | 45 |
| 2B | 310 | 3 | 103 | 0.2 | 21 | 64 |
| 3B | 1025 | 8 | 128 | 0.1 | 13 | 60 |
| 4B | 666 | 8 | 83 | 0.16 | 13 | 56 |
| 5B | 572 | 8 | 71.5 | 0.38 | 27 | 80 |
| 6B | 802 | 8 | 100 | 0.52 | 52 | 55 |
